# Structural characterization of two prototypical repressors of SorC family reveals tetrameric assemblies on DNA and mechanism of function

**DOI:** 10.1093/nar/gkae434

**Published:** 2024-06-06

**Authors:** Markéta Šoltysová, Jana Škerlová, Petr Pachl, Karel Škubník, Milan Fábry, Irena Sieglová, Martina Farolfi, Irina Grishkovskaya, Michal Babiak, Jiří Nováček, Libor Krásný, Pavlína Řezáčová

**Affiliations:** Structural Biology, Institute of Organic Chemistry and Biochemistry of Czech Academy of Sciences, Prague, 166 10, Czechia; Structural Biology, Institute of Organic Chemistry and Biochemistry of Czech Academy of Sciences, Prague, 166 10, Czechia; Structural Biology, Institute of Organic Chemistry and Biochemistry of Czech Academy of Sciences, Prague, 166 10, Czechia; CryoElectron Microscopy and Tomography Core Facility, Central European Institute of Technology, Brno, 601 77, Czechia; Structural Biology, Institute of Organic Chemistry and Biochemistry of Czech Academy of Sciences, Prague, 166 10, Czechia; Structural Biology, Institute of Organic Chemistry and Biochemistry of Czech Academy of Sciences, Prague, 166 10, Czechia; Laboratory of Microbial Genetics and Gene Expression, Institute of Microbiology of the Czech Academy of Sciences, Vídeňská 1083, Prague 142 20, Czechia; Research Institute of Molecular Pathology, Campus-ViennaBiocenter 1, 1030 Vienna, Austria; CryoElectron Microscopy and Tomography Core Facility, Central European Institute of Technology, Brno, 601 77, Czechia; CryoElectron Microscopy and Tomography Core Facility, Central European Institute of Technology, Brno, 601 77, Czechia; Laboratory of Microbial Genetics and Gene Expression, Institute of Microbiology of the Czech Academy of Sciences, Vídeňská 1083, Prague 142 20, Czechia; Structural Biology, Institute of Organic Chemistry and Biochemistry of Czech Academy of Sciences, Prague, 166 10, Czechia

## Abstract

The SorC family of transcriptional regulators plays a crucial role in controlling the carbohydrate metabolism and quorum sensing. We employed an integrative approach combining X-ray crystallography and cryo-electron microscopy to investigate architecture and functional mechanism of two prototypical representatives of two sub-classes of the SorC family: DeoR and CggR from *Bacillus subtilis*. Despite possessing distinct DNA-binding domains, both proteins form similar tetrameric assemblies when bound to their respective DNA operators. Structural analysis elucidates the process by which the CggR-regulated *gapA* operon is derepressed through the action of two effectors: fructose-1,6-bisphosphate and newly confirmed dihydroxyacetone phosphate. Our findings provide the first comprehensive understanding of the DNA binding mechanism of the SorC-family proteins, shedding new light on their functional characteristics.

## Graphical Abstract

**Graphical Abstract:**
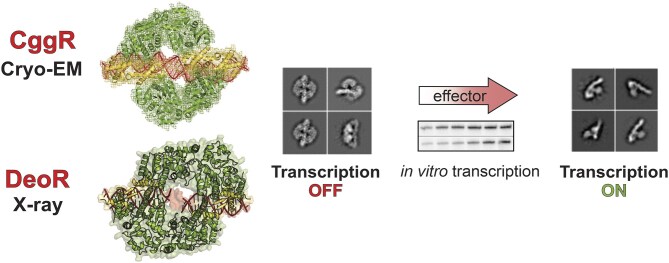

## Introduction

Transcriptional regulation is essential for the rapid and adaptive response of bacteria to the environmental fluctuations they encounter. DNA-binding transcriptional regulators can function as either activators or repressors, facilitating or hindering gene transcription (1). Transcriptional regulators belonging to the SorC family are widespread across all bacteria, particularly of the phyla Proteobacteria (classes α- and γ-Proteobacteria), Firmicutes (classes Bacilli and Clostridia) and Actinobacteria (class Actinomycetia) (2). Currently, the InterPro database contains 8942 proteins with the domain architecture of the SorC family. Their primary role is often to repress carbohydrate catabolism (3–9) and quorum sensing genes (10,11). A typical SorC protein comprises an N-terminal DNA-binding domain (DBD) of a helix–turn–helix (HTH) motif, along with a C-terminal Rossmann-like fold effector-binding domain (EBD) (12–16). The presence of this highly conserved EBD, spanning approximately 250 amino-acid residues, serves as a hallmark of SorC family regulators. The EBD shares sequence homology with glucosamine-6-phosphate deaminases from the NagB/RpiA/CoA superfamily (17,18) and is classified as a sugar-binding domain in the InterPro database (accession No. IPR007324) (2).

SorC repressors generally form tetramers as they bind to their operators located in promoter regions of regulated genes (10,16,19). When a particular effector molecule reflecting the environmental change reaches a ligand-binding site of EBD, the assembly is affected through an allosteric effect (16,19–22). This event leads to the liberation of DNA and the promoter region becomes accessible to RNA polymerase, which initiates gene transcription (23).

There is only limited knowledge about the structural basis underlying the mechanism of action of the SorC family. Previous structural investigations were carried out for individual domains, and only two full-length experimental structures are available today: SorC from *Klebsiella pneumoniae* (12) and LsrR from *Escherichia coli* (15). Efforts have been directed towards understanding ligand recognition and ligand-induced changes (5,6) based on the structural characterization of EBDs from multiple repressors: LsrR from *E. coli* (15) and CggR (13) and DeoR from *Bacillus subtilis* (14). Progress in comprehending DNA recognition by proteins of the SorC family has been achieved recently with structural studies of DBDs of the proteins DeoR and CggR in complexes with their half-operators (24).

**Figure 1.**
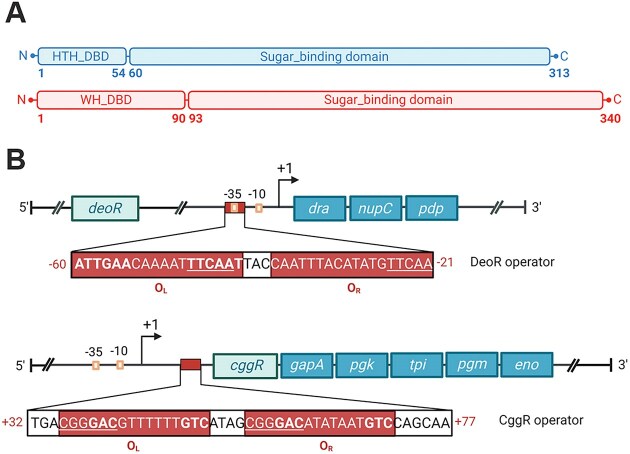
(**A**) Schematic representations of the domain organization of DeoR and CggR from *Bacillus subtilis*. The abbreviations stand for: HTH – helix-turn-helix, WH – winged helix-turn-helix. (**B**) Schematic representations of the operon and operator organization of the *dra–nupC–pdp* (at the top) and *gapA* (at the bottom) operons. The positions of the operators are indicated by red rectangles and the numbers of the positions in red. Palindromic sequences are in bold, and direct repeats are underlined.

Based on two distinct types of DBDs, the SorC family is categorized into two subfamilies: SorC/DeoR and SorC/CggR, named after the above prototypical representatives DeoR and CggR from *B. subtilis* (24,25). The SorC/DeoR subfamily comprises a 50–60 residues long homeodomain-like HTH motif followed by a β-linker, which works as one of the dimerization interfaces (12). By contrast, the SorC/CggR subfamily features a winged HTH (wHTH) motif spanning about 100 amino-acid residues (25) (Figure 1A).

CggR functions as a repressor of the hexacistronic *gapA* operon encoding CggR itself and five crucial enzymes involved in the triose phosphate interconversion during glycolysis (26,27). The operator sequence of 45 bp is located between the +32 and +77 positions relative to the transcription start (21) (Figure 1B). Apo–CggR forms high-molecular-weight oligomers but assembles into tetramers upon binding to the corresponding DNA operator (16). This assembly is composed of a dimer of dimers, each binding half of the operator. Interestingly, the right half of the operator (O_R_) demonstrates 100-fold higher affinity than the left one (O_L_). The CggR–DNA complex was shown to dissociate upon binding of fructose-1,6-bis-phosphate (FBP) (16,21). Additionally, CggR has an affinity for other phosphorylated glycolytic intermediates, such as dihydroxyacetone phosphate (DHAP), glucose-6-phosphate (G6P) or fructose-6-phosphate (F6P) (13).

DeoR acts as a repressor of the *dra–nupC–pdp* operon, which encodes catabolic enzymes for the utilization of deoxyribose and deoxyribonucleoside when other preferred carbon sources are lacking. Deoxyribose-5-phosphate (dR5P) has been reported as the effector molecule for DeoR (19) that binds covalently to the effector-binding site of the EBD (14). The gene for DeoR is located upstream of the operon. In their intergenic region is the operon promoter, which partially overlaps with the operator sequence located between −60 and −21 positions relative to the transcription start site of the operon. The operator sequence is 39 bp long and contains a palindromic region in the promoter upstream side and a direct repeat situated downstream of the −35 hexamer (28). Compared to the operator region of CggR, the binding site of DeoR is not symmetric, however, the left and right half–operators are expected here as well (Figure 1B).

Despite the significance and prevalence of SorC family repressors, a comprehensive understanding of the SorC family function remains incomplete and requires structural insights into the full-length protein-DNA assembly. To bridge this knowledge gap, we focused on DeoR and CggR, two prototypical members of the two SorC subfamilies, and completed structural studies of the proteins bound to their DNA operators, employing X-ray crystallography and Cryo-electron microscopy (cryo-EM). We uncovered the architectural arrangement of the tetrameric protein assembly recognizing its DNA operator. Furthermore, we revealed the mechanism by which the CggR gene undergoes derepression induced by two different effectors and showed DeoR apo-form to be tetramer in solution evincing non-cooperative binding to its operator. Altogether, our findings are a step forward towards a comprehensive understanding of the mechanism of action of the repressors belonging to the SorC family.

## Materials and methods

### Cloning, expression and purification

The genes coding for DeoR (residues 2–313, NCBI sequence NP_391822.1) and CggR (residues 1–340, NCBI sequence NP_391275.1) were cloned into a pET151/D-TOPO vector (Invitrogen, USA) using the Gateway cloning technology (Invitrogen, USA). Each vector included sequences of an N-terminal His_6_ tag, a V5 epitope and a tobacco etch virus protease (TEV PR) recognition site. Upon TEV PR cleavage, the six-amino-acid sequence GIDPFT remained at the N-termini of the recombinant proteins as a cloning artifact.

For structural studies, DeoR and CggR were expressed and purified based on a protocol developed for other bacterial transcription repressors (29). Recombinant proteins were overexpressed in *E. coli* BL21 (DE3) grown on LB broth (Sigma-Aldrich, USA) with the addition of ampicillin at a final concentration of 100 μg/ml. The DeoR and CggR media were further supplemented with glycerol at a final concentration of 0.4% (v/v) and succinate to reach a molarity of 15 mM, respectively. Succinate was used as a carbon source to avoid the binding of glycerol catabolism intermediates by CggR (30).

The bacteria were cultured at 37°C until OD_550 nm_ reached approximately 1. Afterwards, cell cultures were transferred to 18°C and the expression of DeoR and CggR was induced with 0.1 mM and 0.25 mM ethyl-*β*-d-thiogalactopyranoside (ETG), respectively. Cultivation then continued for an additional 14 h.

The bacteria were harvested by centrifugation (4000 × g/20 min/4°C), resuspended in a lysis buffer [50 mM Tris·HCl, pH 7.5, 50 mM NaCl, 5% (v/v) glycerol, 0.02% (*v/v*) *β*-mercaptoethanol, 1 mM protease inhibitor PMSF, 1 μl/ml Benzonase^®^ nuclease (≥250 Units/μl, Sigma-Aldrich, USA) and 2 m*M* MgSO_4_] and lyzed by sonication. The lysate was clarified by centrifugation (40 000 × g/40 min/4°C) and loaded onto a His-select Nickel Affinity Gel (Ni-NTA) column (Sigma-Aldrich, USA) equilibrated in a loading buffer [50 mM Tris·HCl, pH 7.5, 500 mM NaCl, 5% (v/v) glycerol, 0.02% (*v/v*) *β*-mercaptoethanol]. The column was further washed with the loading buffer supplemented with 30 mM imidazole, and the His_6_-tagged proteins were subsequently eluted in one step by the loading buffer containing 350 mM imidazole. In the case of DeoR, the His_6_ tag was then cleaved off by a two-day incubation with the recombinant TEV PR during dialysis against the loading buffer at 5°C. The cleaved His_6_ tag and the TEV PR were removed by reverse Ni-NTA chromatography. The DeoR solution was finally dialyzed against a storage buffer (20 mM trisodium citrate, pH 7.0, 150 mM NaCl, 0.02% (*v/v*) *β*-mercaptoethanol). After the first Ni-NTA chromatography, CggR was subjected to an additional hydroxyapatite chromatography (HApC) step to remove residual DNA. The HAp column (Sigma-Aldrich, USA) was equilibrated in a running buffer [20 mM Tris·HCl, pH 7.5, 50 mM NaCl, 5% (v/v) glycerol, 0.02% (v/v)*β*-mercaptoethanol, 1 mM NaH_2_PO_4_] and CggR was eluted with a gradient of NaH_2_PO_4_ up to a concentration of 250 mM. After HApC, the His_6_ tag was cleaved by TEV PR during a two-day dialysis against the loading buffer at 5°C, followed by reverse Ni-NTA chromatography. CggR was purified further by ion-exchange chromatography (IC) on the Ionex Mono-Q 5/50 GL column (Sigma-Aldrich, USA). The column was equilibrated in IC buffer (20 mM Bis-Tris, pH 6.4, 150 mM NaCl, 0.02% (v/v)*β*-mercaptoethanol), and the loaded protein was eluted by a gradient of NaCl up to a 1 M concentration.

Purified DeoR and CggR (Supplementary Figure S1) were dialyzed into storage buffers (20 mM trisodium citrate, pH 7.0, 150 mM NaCl, 0.02% (v/v)*β*-mercaptoethanol for DeoR and 20 mM Tris·HCl, pH 7.5, 100 mM NaCl and 0.02% (v/v) β-mercaptoethanol for CggR). The composition of the storage buffer was optimized using a differential scanning fluorimetry (Thermofluor) buffer screen (31,32). Finally, the proteins were concentrated on Amicon Ultra concentrators (Millipore, USA) to concentrations in the range of 10–13 mg/ml, aliquoted, and stored at −80°C. The final concentrations were estimated by measuring light absorbance at 280 nm (NanoDrop ThermoScientific, USA) using theoretical absorption coefficients of 0.738 l/(mg · cm) and 0.419 l/(mg · cm) calculated by the ProtParam tool (Expasy) for DeoR and CggR, respectively.

For mass photometry and size-exclusion chromatography experiments, DeoR was overexpressed in *E. coli* BL21 (DE3) grown on minimal medium supplemented with ampicillin at a final concentration of 100 μg/ml and glycerol at a final concentration of 0.4% (v/v). The following procedure was identical with the protocol described above. The DeoR solution was finally dialyzed against a storage buffer (50 mM Tris·HCl, pH 7.5, 150 mM NaCl, 50 mM MgCl_2_, 2 mM EDTA, 0.02% (v/v) β-mercaptoethanol), concentrated on Amicon Ultra concentrators (Millipore, USA) to concentration of 5 mg/ml, aliquoted and stored at −80°C.

### Protein–DNA complexes preparation

All oligonucleotides were purchased from Sigma-Aldrich (USA) as single strands that were hybridized in annealing buffer (20 mM Tris·HCl, pH 7.5, 100 mM NaCl) or TE buffer (10 mM Tris·HCl, pH 8.0, 250 mM NaCl, 1 mM EDTA) by heating to 95°C for 2 min and then cooling down to 25°C in a period of 1 h.

*DeoR*. DNA duplexes used for co-crystallization (Supplementary Table S2) with DeoR were designed based on the physiological operator sequence 5′-**ATTGAA**CAAAAT**TTCAAT**TACCAATTTACATATGTTCAA-3′ (28) (palindromic sequences are in bold, and the direct repeat is underlined). For crystallization experiments, protein–DNA complexes were prepared by mixing DeoR at a concentration of 12 mg/ml with DNA at a molar ratio of 1 : 1.1 and incubated for 30 min on ice. For the purpose of SEC-based DNA binding experiments, DeoR at the concentration of about 2 mg/ml (56 μM) was mixed at a molar ratio of 2 : 1 with O_L18_ and O_R24_, and 4 : 1 with O_LR65_, O_L44_ and O_LR39_ (the sequences are listed in Supplementary Table S1), and incubated on ice for 30 min.

*CggR*. The complete 45 bp operator sequence was used in our cryo-EM studies of the CggR–DNA structure and CggR–DNA state in the presence of selected metabolites: 5′-TGACGG**GAC**GTTTTTT**GTC**ATAGCGG**GAC**ATATAAT **GTC**CAGCAA-3″ (the direct repeats are underlined and the palindromes are in bold), referred to here as O_LR_ (20). The protein-DNA complex was formed by an incubation of CggR at a concentration of 0.86 mg/ml with O_LR_ at a molar ratio of 4:1.5 for 30 min on ice, followed by SEC purification. For the purpose of the cryo-EM analysis, the concentration of the purified CggR–O_LR_ complex was estimated by a Bradford assay (33).

### Size-exclusion chromatography (SEC)

SEC was carried out at room temperature with a Superdex 200 10/300 GL column attached to an Äkta Pure instrument (GE Healthcare, USA) at a flow rate of 0.5 ml/min.

For the purification of the CggR–DNA complex for structural studies, the running buffer contained 20 mM Tris·HCl, pH 7.5, 150 mM NaCl, 0.02% (v/v)*β*-mercaptoethanol, 2 mM EDTA. The column (GE Healthcare USA) was calibrated under the same experimental conditions with phosphate-buffered saline (PBS) as the running buffer. The molecular weight standards purchased from GE Healthcare (USA), were thyroglobulin (669 000 Da), aldolase (158 000 Da), conalbumin (75 000 Da), carbonic anhydrase (29 000 Da), ribonuclease A (13 700 Da) and blue dextran, which was used for the determination of void volume.

For DeoR–DNA binding studies, the running buffer contained 50 mM Tris·HCl, pH 7.5, 150 mM NaCl, 50 mM MgCl_2_, 0.02% *(v/v)*, *β*-mercaptoethanol, 2 mM EDTA. The volumes of loaded samples were 100 μl. The effect of deoxyribose-5-phosphate (dR5P) on the complexes of DeoR–O_LR65_ and DeoR–O_LR39_ was tested by the addition of dR5P to the protein–DNA mixtures (after 30 min of incubation) up to the final concentration of 58 mM and the mixtures were incubated for additional 30 min. The elution profiles were monitored continuously using an online detector at the wavelengths of 280, 260 and 214 nm. The column (Cytiva, USA) was calibrated under the same experimental conditions with the running buffer using the molecular weight standards purchased from Cytiva, USA, namely, thyroglobulin (670 000 Da), γ-globulin (158 000 Da), ovalbumin (44 000 Da) and myoglobulin (17 000 Da). The calibration curve is shown in Supplementary Figure S9H.

### Mass photometry

Measurements of molecular mass of apo-DeoR and apo-CggR were carried out based on a standard protocol (34) on the TwoMP mass photometer (Refeyn, UK). The experiments were conducted using glass coverslips (Refeyn, UK). Silicon gaskets (Grace BioLab, US) were placed on the glass surface to create wells for sample load. A drop of 18 μl of the DeoR storage buffer was used for the autofocus activation. Once focus was identified, 2 μl of the protein solution was added to the buffer, resulting in a final concentration of 30 nM proteins. All samples were measured immediately after quick complex mixing with a 60 s acquisition time using the standard detection area. Mass calibration was done with bovine serum albumin (66, 132 kDa) and IgG (150, 300, 450 kDa) proteins (Sigma-Aldrich, USA). Data acquisition and data analysis were performed using AcquireMP and DiscoverMP software (Refeyn, UK), respectively. Masses of proteins were estimated by fitting the mass histograms with Gaussian distribution.

### Microscale thermophoresis (MST)

MST experiments were performed in the Monolith NT.LabelFree MST instrument (NanoTemper Technologies) using Monolith NT.LabelFree capillaries. The LED (excitation) power and IR-laser (MST) power were set at 20% and 40%, respectively. The analysis was performed using MO.Affinity Analysis v2.3 software (NanoTemper Technologies).

DeoR was measured at the constant concentration of 4 μM. DNA duplexes were titrated by the two-fold serial dilution in following ranges: O_LR65_, 375 μM–11.4 nM; O_L44_, 915 μM–55.9 nM; a negative control (a DNA operator of unrelated transcriptional repressor TreR), 1 mM–30.5 nM. All the samples were prepared in the DeoR storage buffer (50 mM Tris·HCl, pH 7.5, 150 mM NaCl, 50 mM MgCl_2_, 2 mM EDTA, 0.02% (v/v) β-mercaptoethanol). DeoR–O_LR65_ sample was analysed in two independent measurements and a technical triplicate.

### Crystallization of DeoR–DNA

Several DNA variants differing in length and sequence were used for the crystallization of the protein–DNA complex (Supplementary Table S2). DeoR–DNA crystals used for the X-ray structure determination were obtained by co-crystallization with an 18 bp DNA duplex, which was limited to the left half-site of the operator and is referred to here as O_L18_, in line with our previous study (24): 5′-**ATTGAA**CAAAAT**TTCAAT**-3′. The best diffracting crystals were obtained using the hanging-drop vapor diffusion technique manually executed on a NeXtal EasyXtal 15-well plate (Molecular Dimensions, UK) at 18°C and harvested after several months. The DeoR–O_L18_ complex at the final protein concentration of approximately 8 mg/ml was mixed with the crystallization solution at a volume ratio of 1:2 in a total drop volume of 3 μl. The optimized crystallization solution was based on a Nucleix Suite crystallization condition F11 (Qiagen, Germany), and contained: 50 mM sodium cacodylate, pH 6.3, 75 mM CaCl_2_, 25 mM MgCl_2_ and 3.5% (v/v) PEG 2K. The obtained needle-shaped crystals were cryoprotected by quick soaking in 25% *(v/v)* glycerol in the crystallization solution and flash-cooled in liquid nitrogen.

### X-ray data collection, data processing and structure determination of DeoR–O_L18_

Diffraction data were collected at 100 K on the MX beamlines of the Berlin Electron Storage Ring Society for Synchrotron Radiation (BESSY II) light source, operated by the Helmholtz-Zentrum Berlin, Germany (35). The datasets were processed using the XDSAPP software system (36). Data collection and refinement statistics are summarized in Table 1.

**Table 1.** Crystal data and diffraction data collection and refinement statistics

	DeoR–O_L18_
**Data collection statistics**	
Space group	*P*6_5_22
Cell parameters (Å; °)	166.65, 166.65, 331.66; 90, 90, 120
Wavelength (Å)	0.9184
Resolution (Å)	48.93–3.69 (3.91–3.69)
Number of unique reflections	30 268 (4760)
Multiplicity	9,1 (9,7)
Completeness (%)	99.9 (99.9)
*R*_meas_^a^	0.642 (4.378)
CC_(1/2)_^b^	0.992 (0.387)
Average *I*/σ(*I*)	4.93 (0.55)
Wilson *B* (Å^2^)	116.6
**Refinement statistics**	
Resolution range (Å)	48.93–3.70 (3.80–3.70)
Number of reflections in working set	28 152 (1979)
Number of reflections in test set	1482 (104)
*R* value^c^	0.258 (0.464)
*R*_free_ value^d^	0.329 (0.450)
r.m.s.d. bond length (Å)	0.006
r.m.s.d. angle (°)	1.232
Number of atoms in the asymmetric unit	11 264
Mean *B* value (Å^2^)	179.4
Ramachandran plot statistics	
Residues in favored regions (%)	96.7
Residues in allowed regions (%)	3.1
PDB code	8R7Y

Data in parentheses refer to the highest-resolution shell.

^a^ *R*_meas_ – redundancy-independent *R* factor (51).

^b^CC_(1/2)_ is the correlation coefficient between random half datasets; from its value the Pearson correlation coefficient of the true level of signal can be calculated as follows: √2CC_1/2_/1 + CC_1/2_ (52).

^c^ *R* value = ||*F*_o_| − |*F*_c_||/|*F*_o_|, where *F*_o_ and *F*_c_ are the observed and calculated structure factors, respectively.

^d^ *R*_free_ is equivalent to the *R* value but is calculated for 5% of reflections chosen at random and omitted from the refinement process (53)

^e^As determined by Molprobity (48)

The DeoR–O_L18_ structure was determined by molecular replacement with the program Molrep (37,38) of the CCP4 package (39,40). Coordinates of the C-terminal effector-binding domain (EBD) of DeoR (residues 56–313, PDB entry: 4OQQ (14)) and the N-terminal DNA-binding domain (DBD) of the sorbitol operon transcriptional regulator (SorC) from *K. pneumoniae* (residues 1–50, PDB entry: 2W48 (12)) were used as search models for the corresponding domains of the full-length DeoR protein. The sequence identity between the DBDs of DeoR (residues 1–55) and SorC was 48%. The DeoR–DBD was subsequently built into the electron density maps using a model produced by TFmodeller (41). Initial 2*F*_o_ – *F*_c_ electron density maps suggested that two protein dimers and two DNA duplexes were present in the asymmetric unit. Initially, one DNA duplex was located by Molrep when the protein was used as a fixed model and a mutated poly-A/T 18 bp-long DNA fragment from the structure of the diphtheria toxin repressor (DtxR) from *Corynebacterium diphtheriae* (PDB entry: 1F5T (42)) was used as a search model. The nucleotide sequence was subsequently manually mutated in Coot software (43) and the resulting DeoR–O_L18_ complex structure was superposed over the second protein dimer in the asymmetric unit in order to build the second DNA duplex into the electron density map. During the model building process, the high-resolution crystal structure of DeoR_DBD_ in complex with DNA duplex O_15_ (24) became available. This DeoR_DBD_–O_15_ structure was then used to replace the initially modeled DBDs and DNA, constituting a more accurate starting model for further structure refinement. Differences in the DNA sequence and structure were corrected manually in COOT. The Low Resolution Structure Refinement Pipeline (LORESTR) (44) was used during the initial steps of structure refinement. Later refinement stages included manual adjustments in COOT and refinement in REFMAC 5.8.265 with external restrains generated by ProSMART (45). Final refinement was carried out using jelly body and manually generated restrains for DNA duplexes with geometrical parameters suggested by DNATCO (46,47).

The asymmetric unit consisted of two protein dimers and two O_L18_ duplexes. All nucleotides of the two DNA duplexes and protein residues 1–312 were modeled into the electron density map, and the non-macromolecular electron density map was interpreted as eight water molecules. The C-terminal residue (L313) is not present in the crystallographic model because of the absence of electron density, and the N-terminal residue (T1) in chains A, C and D represents a cloning artifact supported by the electron density map. Superposition of the four copies of DeoR in the asymmetric unit revealed negligible differences. The r.m.s.d. values for the superposition of DeoR monomers (chains A and C, 311 Cα atoms) and dimers (chains AB and CD, 622 Cα atoms) are 0.93 and 1.35 Å, respectively.

For structure validation, MolProbity (48) and DNATCO servers were used. The program Superpose of the CCP4 software package (49) was used for structure superposition with homologs. The macromolecular interfaces and inter-molecular interactions were analyzed using the PISA server (50). Two non-interacting dimers each bound to 18 bp DNA double-strands are present in the asymmetric unit (Supplementary Figure S2) and a tetrameric biological unit is formed through interactions with a symmetry-related molecule from the adjacent asymmetric unit (Supplementary Figure S3). The tetrameric biological unit is misaligned because the full operator DNA is represented by two O_L_ fragments, which do not form a continuous DNA duplex that would mimic the full operator. Instead, the DNA is hybridized in an alternative manner in the crystal packing (Supplementary Figure S4), and EBD dimers are thus rotated and shifted relative to the DBD dimers, which is allowed by the flexibility of the linker connecting the DBD and EBD domains. In an attempt to simulate the true conformation of the complex with the complete DNA, we aligned the two 18 bp oligonucleotides and connected them by adding the missing 3 bp in the middle. The DBD–EBD linker was disconnected, and EBD dimers were re-positioned to avoid clashes with the DBD dimers (see Supplementary file DeoR_full_operator_model.pdb).

### Cryo-electron microscopy analysis of the CggR–O_LR_ complex

*Sample preparation*. 3.5 μl of 0.86 mg/ml CggR–O_LR_ was applied to a freshly glow-discharged Cu TEM grid (2/1-C4 C-flat grid, Protochips) and vitrified in liquid ethane using Vitrobot Mark IV (4°C, 100% rel. humidity, 30 s waiting time, 5 s blotting time; Thermo Fisher Scientific, USA). The grids were subsequently mounted into Autogrid cartridges and loaded to Talos Arctica (Thermo Fisher Scientific, USA) transmission electron microscope for screening.

*Data acquisition*. The data were collected using a Titan Krios transmission electron microscope (Thermo Fisher Scientific, USA) operated at 300 kV using the software SerialEM (54). The data were acquired using the post-GIF K2 (Quantum K2) direct electron detector (Gatan, Inc., USA) operating in electron counting mode. The energy-selecting slit was set to 20 eV. Micrographs were collected at a calibrated pixel size of 0.822 Å/px as a set of 40 frames comprising a 5s exposure, resulting in an overall dose of 39 e/Å^2^. The complete dataset consisted of 16818 movies.

*Data analysis*. The movies were first corrected for the drift acquire during data acquisition using the program MotionCor2 (55) and the CTF parameters were estimated using Gctf (56). The images with astigmatism larger than 2000 Å and an estimated resolution based on the CTF fit analysis worse than 6 Å were excluded from further data processing. Micrographs were denoised in the program JANNI (57) using the general model. A set of 20 denoised micrographs was randomly selected from the dataset for manual particle picking using the program e2boxer.py (58). The manually picked particles were used to generate a model for automated particle picking using the program crYOLO (59). A total of 1677668 particles were automatically selected and extracted with a box size of 352 px. Several rounds of reference-free 2D classification on down-sampled particles (box size of 176 px) were used to remove most of the false-positive picks and damaged particles using the program Relion3.1 (60). Selected particles were then imported into the program cryoSPARC (61) and an *ab initio* reconstruction was generated. A non-uniform refinement with C2 symmetry including 220146 particles resulted in the final cryo-EM map at a resolution of 4.3 Å (according to the FSC = 0.143 criterion), used for CggR–O_LR_ data interpretation. Data collection and processing parameters are summarized in Table 2.

**Table 2.** Cryo-EM data collection, refinement and validation statistics

	CggR–O_LR_
**Data collection and processing**	
Magnification	60 386×
Voltage (kV)	300
Electron exposure (e/Å^2^)	39
Defocus range (μm)	−0.5 to −4.0
Pixel size (Å)	0.822
Symmetry imposed	C2
Number of initial particle images	1 677 668
Number of final particle images	220 146
Map resolution (Å)	4.3
FSC threshold	0.143
Map resolution range (Å)	4.5–20
**Refinement**	
Initial model used (PDB code)	2OKG, 7OYK
Model resolution (Å)	4.2/6.7
FSC threshold	0.143/0.5
Map sharpening *B* factor (Å^2^)	−237.6
Model composition	
Non-hydrogen atoms	11 985
Protein residues	1340
Nucleotide residues	82
*B* factors (Å^2^)	
Protein	24.32
DNA	37.60
r.m.s.d. bond length (Å)	0.013
r.m.s.d. bond angles (°)	1.575
Validation	
MolProbity score	1.58
Clashscore	11.70
Poor rotamers (%)	0.18
Ramachandran plot	
Favored (%)	98.34
Allowed (%)	1.66
Disallowed (%)	0
PDB code	8R3G
EMDB code	EMD-18864

### Building of the CggR–O_LR_ model

The cryo-EM map was interpreted using crystal structures of the free CggR_EBD_ (chain A of PDB entry 2OKG (13)) and CggR_DBD_–O_L_ complex (chains A, B, E, F of PDB entry 7OYK (24)) as starting models. In the case of CggR_DBD_–O_L_, the full 20 bp interacting DNA fragment was included into the starting model, composed of the O_L_ 16-mer together with the additional 1 and 4 bp DNA fragments from the neighboring symmetry-related DNA duplexes located at each end of the O_L_ duplex. Occupancy of all residues was set to 1 and minor alternative conformations were removed. The models (EBD monomer and DBD dimer in complex with the 21 bp DNA fragment) were then docked into the map using Phenix 1.16 (62) and the position of each individual DBD and EBD was adjusted by rigid-body fitting in Coot 0.9.1 (43). The DNA fragments were connected (the one extra overlapping base pair was manually removed from the middle of the DNA duplex), mutated to the full operator sequence and refined in Coot using self-restraints for the DNA duplex. DBD cloning artifact residues −4 to 0 were deleted. The DBDs and EBDs were connected and the adjacent regions (residues 87–96 in chains C, B and 87–93 in chains A and D) were refined in Coot with self-restraints. The following EBD regions located at the dimer interface were manually rebuilt and subjected to real-space refinement in Coot: residues 174–179, 183–185, 203–222, 252 and 285–288. Residues 28 in chains C, B and 10 in chains A, D were rebuilt to avoid clashes between the DBDs and EBDs. In contrast to the main chain trace, side chains in the rebuilt regions were not supported by the cryo-EM map and were therefore modeled in non-clashing rotameric positions; identical changes were made in chains C and B, and chains A and D, respecting the C2 symmetry of the cryo-EM map (Supplementary Figure S5). Model quality (Table 2) was assessed in the validation module of Phenix 1.16. The Superpose program of the CCP4 software package was used for structure superposition with homologs. The macromolecular interfaces and inter-molecular interactions were analyzed using the PISA server.

### Cryo-electron microscopy analysis of CggR–O_LR_ in the presence of potential effectors

The CggR–O_LR_ complex was analyzed further by cryo-EM in the presence of known and potential effector molecules, fructose-1,6-bisphosphate (FBP), dihydroxyacetone phosphate (DHAP) and fructose-6-phosphate (F6P). The samples were prepared by mixing the purified CggR–O_LR_ complex at a final protein concentration of 0.86 mg/ml with corresponding concentrations of the ligands and incubated for about 15 min on ice. The final concentrations of F6P, FBP and DHAP were 2 mM, 2 mM and 80 μM, respectively. The concentrations of the ligands were selected based on the expected protein saturation levels while minimizing the image contrast reduction caused by the ligand addition.

*Sample preparation*. 3.5 μl of each sample of CggR-O_LR_ with a ligand were applied to a freshly glow-discharged TEM grid (1.2/1.3-C3 C-flat grid, Protochips) and vitrified in liquid ethane using Vitrobot Mark IV (4°C, 100% rel. humidity, 30 s waiting time, 4 s blotting time; Thermo Fisher Scientific, USA). The grids were subsequently mounted into Autogrid cartidges and loaded into a Talos Arctica transmission electron microscope (Thermo Fisher Scientific, USA) for data acquisition.

*Data acquisition*. The CggR–O_LR_–FBP, CggR–O_LR_–F6P and CggR–O_LR_–DHAP data were collected on a Talos Arctica microscope operated at 200 kV, using the software SerialEM (54). Micrographs were acquired at a calibrated pixel size of 1.23 Å/px with a Falcon 3EC direct electron detection camera (Thermo Fisher Scientific, USA) operating in charge integration mode. The data from 1.0 s exposure using an overall dose of e/Å^2^ were split into 40 frames. The dataset of CggR–O_LR_–FBP, CggR–O_LR_–F6P and CggR–O_LR_–DHAP comprised 1386, 1476 and 842 movies in total, respectively.

*Data analysis*. The software and parameters for the movie correction and particle picking were set the same as in the case of CggR–O_LR_. A total of 388 109, 711 657 and 225 436 particles were automatically selected from the CggR–O_LR_–FBP, CggR–O_LR_–F6P and CggR–O_LR_–DHAP datasets, respectively. Particles were extracted with a box size of 256 px and down sampled to a box size of 128 px. Several rounds of reference-free 2D classification were used to remove most of the false-positive picks and damaged particles using the program Relion3.1 The cryo-EM data for CggR–O_LR_ in the presence of effectors were interpreted at the level of 2D classes, and the selected 2D classes comprised 64 247 particles for CggR–O_LR_–FBP, 67 731 particles for CggR–O_LR_–F6P and 42 293 particles for CggR–O_LR_–DHAP. The selected negative control 2D classes of CggR–O_LR_ originated from the Titan Krios dataset described above and comprised 43 633 particles.

### DNA manipulation for *in vitro* transcription assay of CggR

A 133 bp long promoter fragment (Supplementary Figure S6) from the upstream region of the *cggR* gene was PCR-amplified (Roche Expand) with the primers PG-3R and PG-4aH (EastPort, Czechia) containing introduced EcoRI and HindIII restriction sites. The fragment was cloned *via* these restriction sites into the p770 plasmid (63), yielding the construct LK646, which was subsequently validated by sequencing.

- PG-3R: 5′-ggAATTCTTTTggCTATgACgggACg-3′
- PG-4aH: 5′-gCCAAgCTTCTggTTCATgACTCAAACgTTCC-3′

### Preparation of RNA polymerase (RNAP) holoenzyme for *in vitro* transcription assay of CggR

*Bacillus subtilis* RNAP, His_10_-tagged on the β' subunit, was purified from strain MH5636 as described (64). The σ^A^ of RNAP was overproduced from plasmid pCD2 (65) and purified as described (66). Core RNAP and σ^A^ were reconstituted at a molar ratio of 1:5 in storage buffer (50 mM Tris·HCl pH 8.0, 0.1 M NaCl, 0.1 mM DTT, 50% (v/v) glycerol) for 30 min at 30°C.

### *In vitro* transcription assay of CggR

Transcription was carried out as described in (67) but with several modifications. Multiple-round transcription was performed in 10 μl reactions with 30 nM RNAP, 6.5 nM supercoiled plasmid template, 40 mM Tris·HCl pH 8.0, 10 mM MgCl_2_, 1 mM DTT, 0.1 μg/ml BSA, and 55 mM NaCl. ATP was 800 μM, GTP 500 μM, CTP 200 μM and UTP was 10 μM plus 2 μM [α-^32^P] UTP. Known or potential inducers (dihydroxyacetone phosphate – DHAP, fructose-1,6-bisphosphate – FBP, fluctose-6-phosphate – F6P, glucose-6-phosphate – G6P; Sigma-Aldrich, USA) and a negative control glucose-1-phosphate (G1P; Sigma-Aldrich, USA), were used serially diluted, from 1 mM down to a final concentration of 0.03125 mM in 2-fold dilution steps. CggR was used at a constant concentration of 240 nM. Reactions were pre-incubated for 5 min at 30°C, initiated with RNAP, allowed to proceed for 15 min at 30°C, terminated by the addition of an equal volume of formamide loading buffer (95% formamide, 20 mM EDTA pH 8.0), electrophoresed on 7 M urea 7% polyacrylamide gels, and quantified by phosphorimaging (Amersham Typhoon, Cytiva, USA).

## Results

### DeoR tetramer binds two O_L18_ half-operators in the crystal

**Figure 2.**
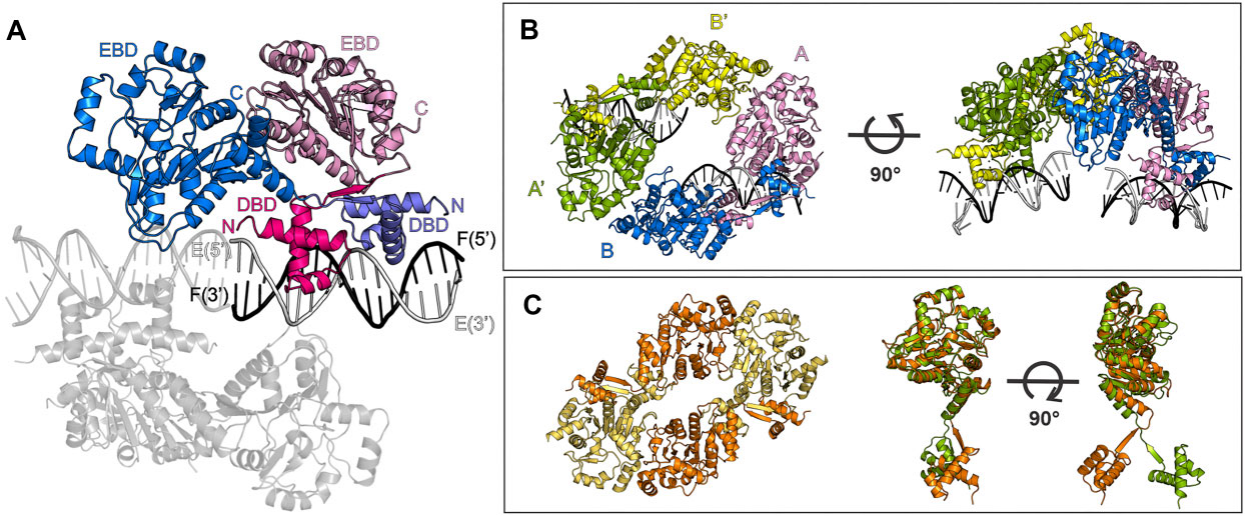
Crystal structure of DeoR–O_L18_: the asymmetric unit and the biological unit. (**A**) The asymmetric unit contains two equivalent DeoR dimers bound to O_L18_. One copy of this complex is described using labels and colors, and the other is in grey. The labels stand for: EBD – effector-binding domain, DBD – DNA-binding domain, C – protein C-terminus, N – protein N-terminus, E (5′/3′) and F (5′/3′) – DNA strands with indicated ends. (**B**) The tetrameric assembly consists of two symmetry-related asymmetric units. The protein monomers of the symmetry-related dimer (A′ and B′) are colored green and yellow, respectively. (**C**) On the left, the structure of a homologous tetrameric assembly of apo-LsrR from *E. coli* (PDB entry 4GO1 (20)) is shown; on the right, the superposition of monomers of DeoR and LsrR.

To understand the molecular mechanism of how DeoR interacts with its DNA operator, we attempted to obtain the crystal structure of DeoR in complex with DNA. To ease the crystallization, we used various DNA constructs based on the sequence located between positions −60 and −21 relative to the transcription start point of the *dra–nupC–pdp* operon (28) (Supplementary Table S2). Successful crystallization was achieved for a complex with an 18 bp long DNA duplex (referred to here as O_L18_) that contained the first two palindromic binding sites of the operator (5′-**ATTGAA**CAAAAT**TTCAAT**-3′), specifically the left-half operator sequence. The crystal structure was determined by molecular replacement and refined to a resolution of 3.7 Å (Table 1). The asymmetric unit comprised two physiological protein dimers, each bound to one O_L18_ duplex (Figure 2A). All nucleotides and protein residues 1–312 were modeled into an electron density map; the non-macromolecular electron density map was interpreted as eight water molecules.

Each monomer within the dimer contains an N-terminal HTH-type domain (24) and a C-terminal effector-binding domain of a three-layer (α/β/α) sandwich architecture containing a double Rossmann fold (14). The two domains are connected through residues 53–57 following the β1 strand in the DBD. The antiparallel β-sheet formed upon DBD dimerization results in swapped positions of the EBD domains with respect to the N-terminal DBDs (Figure 2A). The dimeric interface is provided only by the N-terminal domains; interactions of the C-terminal domains within the dimer are negligible.

The arrangement of the antiparallel β-sheets places the C-terminal EBDs into swapped positions in which they do not interact with each other but are capable of further association with EBDs from other dimers. Within the crystal, this interaction was observed between two symmetry-related DeoR dimers originating from the neighboring asymmetric unit (Supplementary Figure S3). This tetramer, whose architecture can be described as a dimer of dimers, is depicted in Figure 2B for chains A and B and symmetry-related chains B′ and A′. Structurally, the DeoR protein is very similar to LsrR from *E. coli* whose structure was determined by X-ray crystallography (PDB entry 4GO1) and shows that LsrR also assembles into tetramers (20). Comparison of the two structures revealed differences in mutual orientations of EBD and DBD domains, which might be caused by the fact that LsrR was crystallized in its free form (Figure 2C).

The total surface area buried upon the formation of the tetramer is 655 Å^2^ for the interface mediated through DBDs and 1301 Å^2^ for the interface mediated through EBDs, representing 4% and 8% of the total accessible surface area of a full-length monomer, respectively. The interaction interface between EBDs had been previously described in the structure of truncated DeoR containing only the EBD domain (PDB entry 4OQQ). In this context, the isolated EBD formed dimers in essentially the same arrangement and exhibited interaction interfaces consistent with those observed in the full-length protein (Supplementary Figure S12A). Furthermore, the interaction interface is located in the vicinity of the effector-binding site and is indeed affected by the interaction with the effector, deoxyribose-5-phosphate (14).

The DNA is in the B-form with a majority of the dinucleotide steps belonging to the BBB group of the Conformational Alphabet of Nucleic Acids (CANA), as assigned by DNATCO (46). The DNA duplexes in the crystal form a pseudo-continuous double helix through head-to-tail packing. This packing is possible thanks to the hybridization of the adjacent DNA strands and stabilized further by EBD residues contacting the adjacent DNA duplex (Supplementary Figure S2).

The DeoR dimer interacts with the major DNA groove and the DBDs within each dimer are spatially separated by about one DNA turn (Figure 2A). The interface area between the DBD dimers and DNA duplex is 1042 Å^2^, accounting for approximately 6.4% of the total DBD dimer solvent-accessible surface area, and the protein–DNA interactions (Supplementary Figure S7) are strongly similar to those captured in the high-resolution structure of the separate DeoR DBDs bound to a 15-bp DNA duplex (DeoR_DBD_–O_15_) (24). The O_15_ oligonucleotide (5′-**TTGAA**CAAAA**TTCAA**-3′) used in the previous work was also based on the left palindromic part of the operator. However, it differed from O_L18_ in that it lacked a base pair at each terminus and a TA base pair located at the 3′ end of the inter-palindromic region.

The symmetrical distribution of the interactions with the phosphates in the DNA backbone corresponds to that found in the DeoR_DBD_–O_15_ structure. The same is true for the base-specific interactions in the left part of the O_L_ half-operator (5′-**TTGAA**CA-3′), except for the missing interaction between R39 and T14 in the full-length structure. Nonetheless, differences in base-specific interactions are observed in the right-part palindrome region neighboring the inter-palindromic linker's TA base-pair, which was absent in the DNA variant in the DeoR_DBD_–O_15_ structure.

The major discrepancy lies in the interactions of residue R39. In DeoR_DBD_–O_15_, R39 (chain B) interacts only with the sugar-phosphate backbone of the DNA from the adjacent asymmetric unit whereas in DeoR–O_L18_ R39 contacts the bases of T14 and G4. In addition, residue R34 interacts with the base of T12, the very nucleotide missing in the DeoR_DBD_–O_15_ model (Supplementary Figure S7). Although the limited resolution of the DeoR–O_L18_ crystal structure does not allow observing protein-DNA interactions with high confidence, they might be anticipated based on the previously published high-resolution DeoR_DBD_-O_15_ structure. Moreover, the sidechains proposed to be interacting with DNA are highly conserved within the SorC/DeoR subfamily (Supplementary Figure S8) and they are in the sufficient vicinity with bases which were shown to be essential for the protein binding (28).

### A tetrameric assembly represents a biological unit of DeoR

Although the crystallographic asymmetric unit contained two independent DeoR dimers, interaction analysis within the crystal lattice revealed a tetrameric assembly that is likely to represent a biologically relevant unit (Figure 2B). Previous research has demonstrated that the DeoR indeed forms tetramers in the free form (14,19) and tetrameric assembly upon DNA binding was observed for other members of the SorC family, such as CggR (65) and for LsrR (10).

To investigate oligomeric state and affinity of DeoR to DNA duplexes representing various parts of DNA operator in solution, we combined size-exclusion chromatography (SEC), mass photometry and microscale thermophoresis (MST). SEC was performed for DeoR mixtures with five alternative DNA duplexes (Supplementary Figure S9). Analysis confirmed protein–DNA complex formation only for duplexes containing the full operator sequence in the context of 65 bp long oligonucleotides as well as the minimal 39 bp operator sequence (Supplementary Figure S9A, B). None of the individual half-operators evinced affinity to DeoR and neither did the 44 bp long fragment containing the left palindrome flanked by adjacent regions but missing the 3′ direct repeat (Supplementary Figure S9C–E). We assume that the complex of DeoR and O_L18_ was formed due to the high concentration of the binding partners in the crystallization drop.

Mass photometry confirmed that the DeoR molecules assemble into a tetrameric population (Supplementary Figure S10). Protein was investigated at nanomolar concentration, which is closer to concentrations observed in the living cell (68), and we might conclude that tetramer represents a physiological state of DeoR recognizing its DNA operator. Our attempts to determine the mass of the DeoR–DNA complex were not successful as molecular weight did not change upon addition of 65 bp DNA operator sequence. (Supplementary Figure S10). This could be explained by the fact that DeoR affinity to the DNA is lower than expected (19), and we are not able to capture any population corresponding to a DeoR-DNA complex at nanomolar concentrations required by the method. Indeed, MST (69,70) analysis of the DeoR interaction with the 65 bp-long O_LR_ provided the *K*_D_ value of about 30 μM (Supplementary Figure S11). The Hill's coefficient value slightly lower than 1 suggests that the interaction is not cooperative and binding of the DeoR tetramer to the full operator sequence is expected.

Combination of the above-described results approve that DeoR tetramer assembled on two half-operators observed in crystal can be used as a structural model for biologically relevant tetramer binding full-length operator.

### Cryo-EM structure of CggR bound to the full operator

To understand the molecular mechanism of how CggR interacts with its DNA operator, we employed single-particle cryo-electron microscopy (cryo-EM). We determined the structure of CggR bound to the complete DNA operator, referred to here as O_LR_. The DNA duplex O_LR_ contains the full-length operator sequence of 45 bp, located between positions +32 and +77 relative to the transcription start point of the *gapA* operon (21).

Our 2D particle classification indicates that, when bound to DNA, CggR forms a tetrameric assembly with a global C2 symmetry (Supplementary Figure S13A). The C2 symmetry was clearly apparent also in the reconstructed map refined without enforcing any symmetry, and the final 3D reconstruction using 220146 particles was therefore refined with C2 symmetry to a resolution of 4.3 Å (Supplementary Figures S5, S13, statistics in Table 2). Despite the limited resolution, we were able to build a nearly complete model of the full biological unit of CggR, thanks to the previously published high-resolution structures of the EBD and DBD bound to a 16 bp long half-operator (13,24), which we docked into our cryo-EM map. The local resolution of the map did not allow for the modeling of the two terminal base pairs at each end of the DNA duplex. The protein model comprises residues 1–338, but residues 180–182 in a surface-exposed loop in the EBD are not modeled, as they were also missing in the docked X-ray structure.

**Figure 3.**
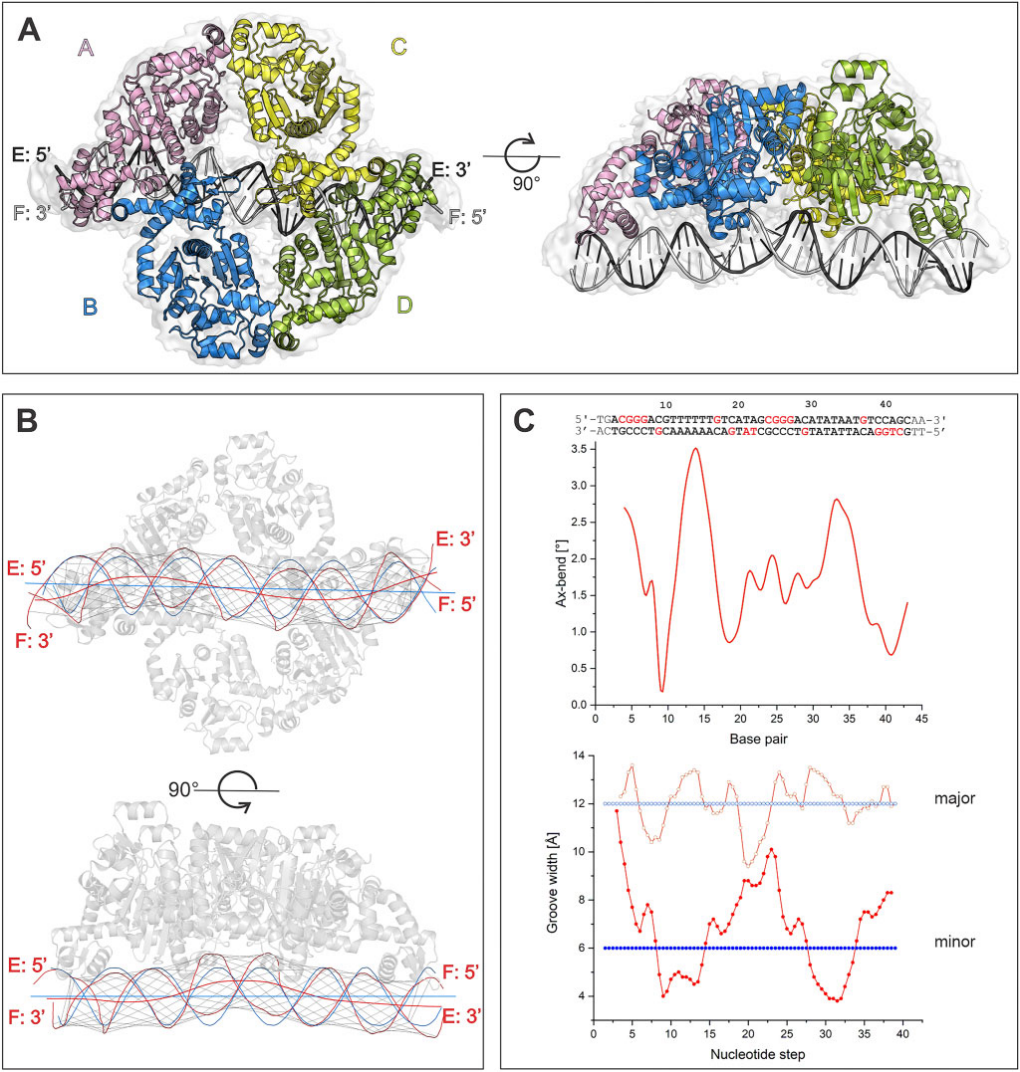
DNA binding to CggR in the cryo-EM structure. (**A**) Model of the CggR–O_LR_ biological unit fitted to a cryo-EM map. (**B**) Visualizations of the helical axis of the O_LR_ DNA bound to CggR, calculated with the software Curve+. The blue lines represent the course of an ideal B-DNA molecule, and the red lines represent that of the O_LR_ DNA in the cryo-EM structure. The thin black lines depict the vectors defining the major and minor groove widths in the O_LR_ DNA. (**C**) Plots of the angular course of DNA axis bending (Ax-bend, at the top) and groove widths (at the bottom) as calculated using Curve+ software. The major and minor groove widths of ideal B-form DNA are drawn as blue lines. Both plots are aligned with the O_LR_ operator sequence. Nucleotides contacted by the protein are highlighted in red. The first and last two base pairs are not in bold because they were not modeled into the cryo-EM map.

As anticipated based on the previous investigations (16,22), CggR binds to DNA as a tetramer. Each monomer of CggR contains an N-terminal two-β-stranded wHTH motif with an extension of a C-terminal helix (24) followed by a C-terminal effector-binding domain that shares great homology with other members of the SorC family (13,14). Despite the presence of diverse DNA-binding domain types, the architecture of the CggR–O_LR_ complex bears a striking resemblance to the DeoR–DNA biological unit described above. In this configuration, each half-operator is recognized by a CggR dimer. The dimerization is mediated through DNA-binding domains, predominantly through a swap of the N-terminal α1 helices. The connection between the DBD and EBD in CggR is made by a short region (residues 92–94) between two helices and, in contrast to DeoR, the arrangement of EBDs does not involve domain swapping. Two CggR dimers come together to form a tetrameric assembly via an interaction between the EBDs of the two neighboring dimers (Figure 3A). The interaction interface between the EBDs had previously been elucidated in the structure of CggR containing the EBD domain only (PDB entry 2OKG) (13). Nevertheless, a comparison revealed certain differences between the X-ray and cryo-EM structures. Notably, significant local differences in the main chain trace were observed at the interface between the two EBDs in the dimer, particularly in helix α10 and the surrounding loops (Supplementary Figure S12B). These regions are known to undergo rearrangements upon an effector binding to the effector-binding site (13). Furthermore, differences in the mutual orientation of EBDs become apparent when the dimers are aligned (Supplementary Figure S12B). Consequently, there is a difference in average EBD–EBD dimer interface area in the cryo-EM structure (649 Å^2^) compared to the X-ray structure (771 Å^2^; Supplementary Figure S12B). We assume that the difference in the dimerization angle between the EBDs is likely a result of context in the full-length protein–DNA complex and that the cryo-EM structure is likely the more accurate representation of an authentic native state of the repressor–DNA complex compared to the high-resolution crystal structures of the dissected domains.

The interaction of CggR with DNA is mediated by DBD dimers, each interacting with one half-operator, wedging into two consecutive major grooves. The DBDs of chains B and C, which are closer to the center of the operator, are separated by about one DNA turn (Figure 3A). Comparison between the structure of individual DBD dimers with the high-resolution crystal structure (PDB entry 7OYK), reveals no difference in the main chain trace, except for the C-termini, linking the DBDs to the EBDs. The alignment of dimers also uncovers a minor difference in the angle between the two DBDs in the dimer (Supplementary Figure S12D).

The conformation of the operator DNA is significantly affected by CggR binding (Figure 3B, C) and exhibits a profound bend in the regions contacted by DBD dimers. Specifically, Curve + software analysis (71) revealed the most significant axis bend deviations from the ideal B-form DNA in the minor groove of the poly-AT region of the left half-operator and the minor groove of the TATA region of the right half-operator, which are located between the interacting DBD monomers. A similar DNA geometry has been observed in the high-resolution crystal structure of CggR_DBD_–O_L_ (24). Poly-AT/TA regions are frequently found to contribute to DNA distortion in general (72,73), and their presence between the individual binding sites of O_L_ and O_R_ likely imparts local plasticity to the DNA, thereby facilitating repressor binding. For example, analogous compression of the minor grooves upon protein binding has been observed in the structure of Controller protein–DNA complexes (74). Another noteworthy feature of the bound DNA is the apparent kink in the middle of the DNA duplex, where the central minor groove, flanked by the DBD dimers, is substantially widened (Figure 3C). The dinucleotide steps in this central DNA region adopt an A-conformation, as assessed by the DNATCO server. The conformational change from B- to A-form DNA in bent regions of the protein-bound DNA is well studied (75) and has also been observed in DNA complexes of other transcriptional regulators (76,77).

Despite the low resolution, integrating the full-length cryo-EM model with a previously published high-resolution structure of CggR DBD bound to the 16 bp long left part of the operator (CggR_DBD_–O_L_) (24) provides insight into the how the repressor interacts with the previously uncharacterized right operator region O_R_. The interface areas between the DBD dimers and the O_L_ and O_R_ of the operator are nearly identical (1412 Å^2^ versus 1376 Å^2^). Whereas the contact points between the DBD dimers and the DNA phosphate backbone remain conserved between O_L_ and O_R_ (Supplementary Figure S14), the base-specific interactions differ due to variations in the sequences of the half-operators. Considering the limited resolution of the cryo-EM map, we can only speculate how the different sequences of O_L_ and O_R_ may influence base-specific interactions. In the left part of O_R_, the sequence matches that of O_L_, so we anticipate analogous base-specific interactions also confirmed by the high-resolution structure. Specifically, three arginine residues (R38, R37 and R52) interact with three consecutive guanines in DNA strand E and arginine R49 contacts a guanine in strand F. However, because the sequences in the right parts of the half-operators differ, we can only propose interactions between R37, R49 and R52 with guanine bases in O_R_ based on analogous interactions observed in the high-resolution structure of the O_L_ (Supplementary Figure S14).

### Transcription derepression captured by cryo-EM and the *in vitro* transcription assay

We performed cryo-EM analysis of CggR–O_LR_ in the presence of selected intermediates of the central glycolytic pathway that is under the control of the CggR regulator: fructose-6-phosphate (F6P), fructose-1,6-bisphosphate (FBP) and dihydroxyacetone phosphate (DHAP). All these compounds have been shown to have an affinity towards the CggR EBD (13); however, only some are considered genuine effector molecules. The effector role of FBP was established by a variety of biophysical studies (73) whereas the effector role of DHAP was merely suggested based on the highest affinity towards CggR and the negative effect on the DNA binding observed in the electro-mobility shift assay (13). Our objective was to capture the release of CggR from the DNA operator upon the addition of these molecules to validate their roles as effectors.

**Figure 4.**
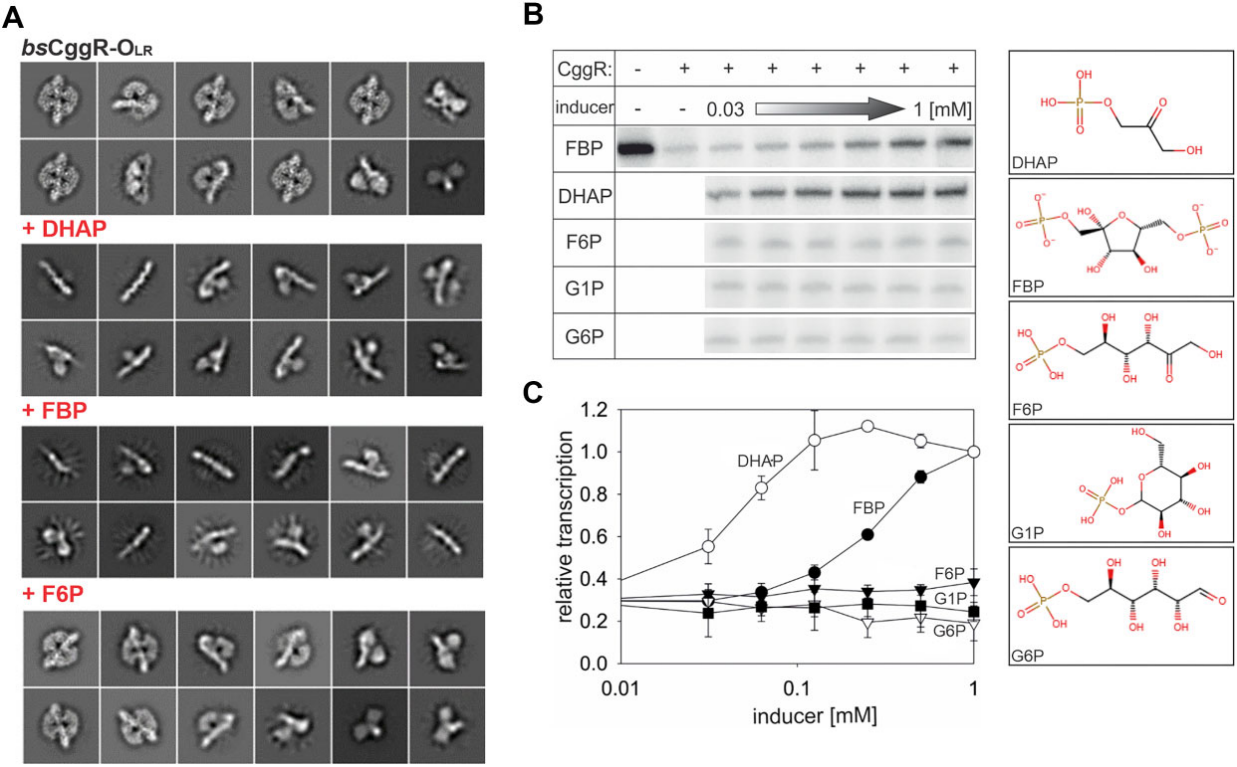
Identification of true effectors of CggR. (**A**) cryo-EM analysis of the CggR–O_LR_ complex in the presence of selected glycolysis metabolites. Representative 2D particle classes of CggR–O_LR_ alone and in the presence of the known or potential effectors FBP, DHAP or F6P. While CggR with no added carbohydrate and upon addition of F6P remains intact, the presence of FBP and DHAP clearly show the disintegration of the particle. (B, C) *In vitro* transcription assay. Multiple-round *in vitro* transcription from the P*_CggR_* promoter was carried out in the presence of CggR with increasing concentrations of FBP, DHAP, F6P, G1P or G6P. (**B**) Examples of primary data – radioactively labeled transcripts resolved on a polyacrylamide gel visualized by phosphorimaging. The concentration range of the inducers tested spanned from 1 mM down to 0.03 mM in two-fold dilutions steps. (**C**) Quantification of the *in vitro* transcription experiments: DHAP – empty circles; FBP – filled circles; F6P – filled triangles; G1P – empty triangles; G6P – filled squares. The data points are averages of at least three biological replicates and the error bars represent the range of standard deviations (±SD). Transcription at 1 mM DHAP was set as 1. The basal level of relative transcription (in the presence of CggR and the absence of any metabolite) was ∼0.3 (not shown).

The analysis of 2D classes revealed intact protein–DNA complexes in the presence of F6P, where DNA-bound tetrameric protein particles similar to the control ligand-free experiment were observed. In the presence of DHAP or FBP, our 2D classes document various stages of the disassembly of these complexes (Figure 4A). The results demonstrate that the addition of DHAP and FBP clearly causes the release of the protein from the DNA and thus leads to transcription derepression. On the other hand, F6P does not have any observable effect on the repressor's affinity towards DNA.

In a complementary experimental approach, *in vitro* transcription from the P*_CggR_* promoter was observed in the presence of CggR and one of the carbohydrates, DHAP, FBP or F6P. Two additional phosphorylated sugars from the glycolysis pathway were also used in the assay: glucose-6-phosphate (G6P), which was tested as a potential effector (13), and glucose-1-phosphate (G1P), which was used as a negative control. The results show that only two of the tested glycolysis metabolites, FBP and DHAP, induce transcription from P*_CggR_*. DHAP triggers an *in vitro* transcription response even at lower concentrations than FBP, confirming that DHAP functions as a true effector molecule of CggR (Figure 4B, C).

## Discussion

Transcriptional regulators of the SorC family are essential for the control of the carbohydrate metabolism and quorum sensing. However, the molecular mechanisms of their action are still largely unexplored. CggR and DeoR from *B. subtilis* are two prototypical representatives of the family which have lent their names to two subfamilies recognized within it. Here, we present two three-dimensional structures of the full-length proteins bound to their DNA operators and elucidate mechanisms by which these SorC representatives repress transcription. Additionally, we visualize and describe the process of derepression of CggR upon the addition of a small-molecular effector. Based on structural studies of these two representatives, we outline molecular mechanisms that might be used for evaluation of other transcriptional repressors of the SorC family.

**Figure 5.**
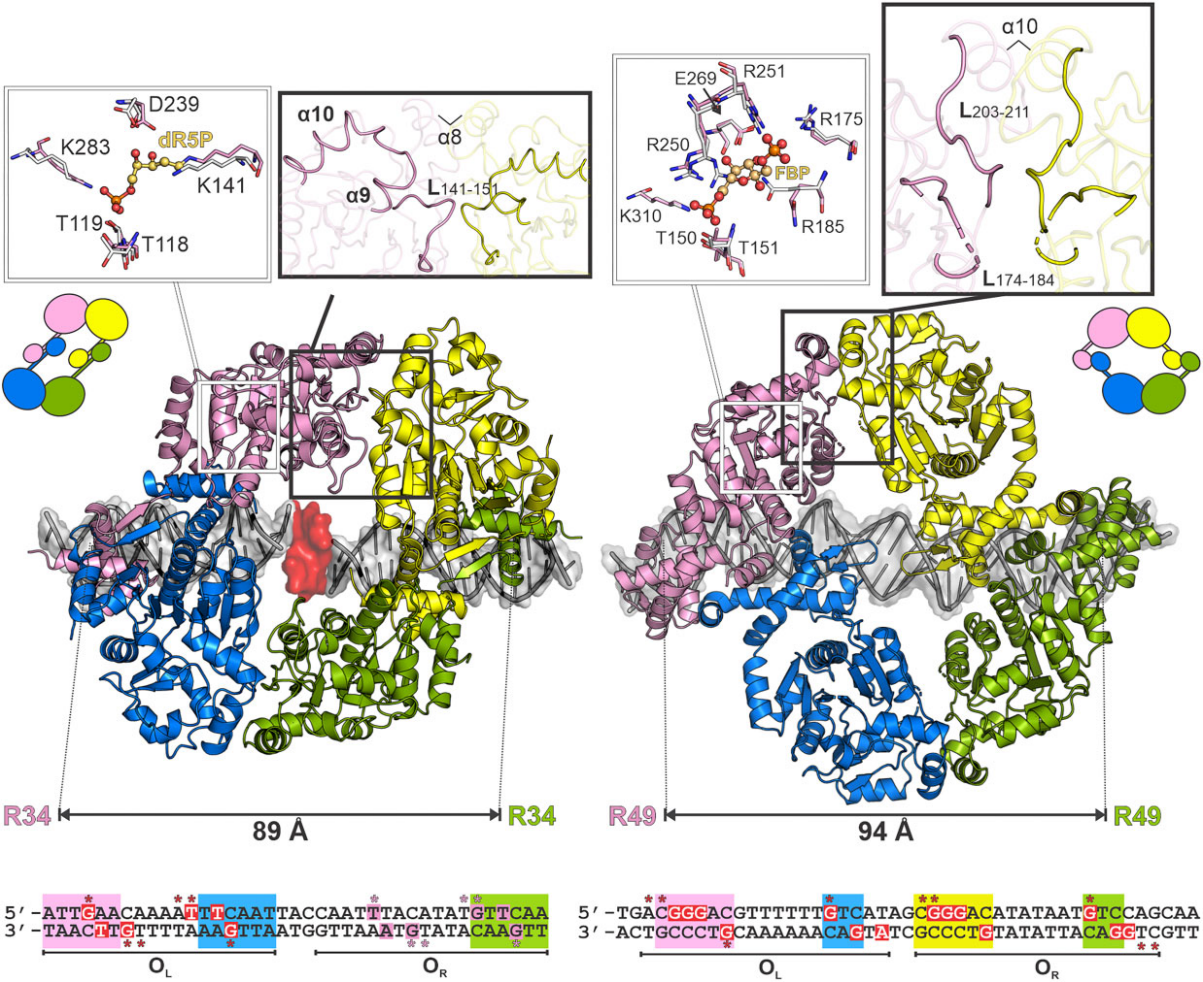
Comparison of full-length DNA complexes of DeoR (left) and CggR (right). The most crucial conformational differences upon the effectors binding are highlighted in the thick-outlined zoom-in boxes. The superpositions of effector-binding sites are shown in the thin-outlined zoom-in boxes. The residues of the DNA-bound (non-liganded) and effector-bound forms are shown as pink and white sticks, respectively. The effector molecules, dR5P and FBP for DeoR and CggR, respectively, are shown as balls and sticks with carbon atoms in pale orange. The distances between the most distant DNA-binding residues are indicated. At the bottom, the sequences of DNA operators are presented with highlighted palindromic and direct-repeat regions in rectangles of the same color code as the corresponding contacting DBDs. Protein-interacting nucleobases and phosphates are depicted in red rectangles and labeled with asterisks, respectively. In the case of DeoR, the proposed interaction pattern in O_R_ is shown in pink.

Structural information obtained by two experimental techniques for two SorC members proves the formation of a tetramer upon binding to a DNA operator sequence. Our cryo-EM study of CggR demonstrates that the protein in complex with its 45 bp DNA operator forms particles consisting of protein tetramers exhibiting global C2 symmetry (Figure 4A). The crystal structure of the DeoR–DNA complex revealed that the DeoR dimer interacts with the 18 bp DNA half-operator. A tetrameric assembly is formed through the interaction of EBDs with the dimer from the neighboring asymmetric unit in the crystal lattice, and this crystallographic tetramer allowed us to construct our biologically relevant model of DeoR bound to the full operator (Figure 5).

The SorC tetrameric architecture can be described as a dimer of dimers. First, a dimer is formed through an interaction between two N-terminal DBDs, and a tetrameric assembly is achieved when these two dimers dimerize together through C-terminal EBDs. The formation of a tetrameric assembly bound to DNA operator had previously been proposed for CggR (16,21,78). Our cryo-EM analysis revealed the exact molecular architecture of the tetramer, which was not previously captured by the small angle X-ray scaterring (SAXS) studies (16).

CggR and DeoR each have different DBDs that classify them into two SorC subfamilies. Additionally, there are also differences in the operator sequences and their respective location. Nevertheless, despite these differences, both operators are composed of two halves containing signatures for recognition by DNA-binding domains. In the case of CggR, two conserved palindromic sequences were previously identified within its operator (21) whereas previous studies of the DeoR operator sequence recognized only the palindrome on the left side of the operator and a direct repeat on the right (28). Based on our results, we propose the existence of an additional cognate sequence in the right half-operator, which was not recognized previously because it differs from the conserved pattern TTCAA (Figure 5). Interaction with any of the two half-operators was not confirmed by size-exclusion chromatography (Supplementary Figure S9) suggesting lower affinity for DeoR, and the interaction may only occur through avidity effect promoted by interaction of DeoR tetramer with the O_L_ palindrome and the O_R_ direct repeat (16,20,24,28,78). In this arrangement, DeoR interacts with sequence TTACA located upstream from the direct repeat of the left half-operator (Figure 5). Although the base-specific contacts within the right part can be expected, we assume that interaction with phosphates is especially important in this region. The high-resolution structure of DeoR_DBD_ previously revealed role of equally distributed interaction with the phosphate backbone, which was able to substitute the base-specific contacts (24).

The similar arrangement of signature sequences within the CggR and DeoR operators allows for the conserved positioning of their DNA binding domains when bound to DNA. Even though each SorC representative possesses a domain from a different subfamily, both include a helix–turn–helix motif variation and form dimers. These dimers further interact with each other in the SorC family's conserved mode through their C-terminal EBDs, which results in a tetrameric assembly formation. The tetramers recognize their operators in an orientation to the same side of DNA in an analogous arrangement, as demonstrated by similar distances between the outer DNA binding residues of CggR and DeoR (Figure 5). Each of the SorC proteins explored here employs a distinct strategy for DBD dimerization, and this results in a different mutual orientation of the N- and C-terminal domains within the tetramer. CggR forms the DBD dimerization interface through N-terminal α1 helices whereas DeoR dimerizes through C-termini of DNA-binding domain, forming an antiparallel β-sheet, and this results in the swapping of the position of the C-terminal domains. Nonetheless, despite this difference in the dimerization mechanism, the global shape of both SorC-family repressor assemblies is closely similar, and the EBDs contact each other via a conserved interface (Figure 5).

Despite the similar tetrameric architecture achieved upon DNA binding, the mechanisms of DeoR and CggR interaction with their operators are different. For CggR, cooperative binding of two dimers to a DNA operator had previously been proposed (16,20,65) while DeoR binds the DNA directly as a tetramer. In this work we confirmed tetrameric assembly of DeoR at low concentrations in solution (Supplementary Figure S10) and the value of the Hill coefficient of approximately 0.9 derived from the MST binding isotherm suggests non-cooperative binding of protein to DNA (Supplementary Figure S11).

Each of the dimeric interfaces within the tetramer serves a role in one of the two critical aspects of repressor function: DNA recognition and response to an effector molecule. Dimerization of the DBDs is essential for the specific recognition of the DNA operator. The formation of dimers of the EBDs physically bridges two DNA binding events and enhances the interaction with DNA sites through an avidity effect. At the same time, the dimerization interface of EBDs is a region that responds to structural changes induced by a ligand binding into the effector-binding site. The EBDs of CggR and DeoR, as well as the other members of the SorC family, are phosphosugar-binding domains homologous to glucosamine-6-phosphate deaminases from the NagB family (25). The active site is adapted to bind effectors, and in the cases of CggR and DeoR, these effector molecules are intermediates of controlled metabolic pathways.

Ligand-induced structural changes had previously been demonstrated in crystallographic models of individual EBDs in both CggR and DeoR (13,14). These conformational changes are most probably propagated to the interface α-helix 8 and 10 in DeoR and CggR (Figure 5), respectively, leading to the destabilization of the dimeric interface or propagate further trough allosteric effect to the DBDs.

By leveraging the capabilities of cryo-EM, we were able to observe the disassembly of the CggR tetramer from the DNA operator (Figure 4A). We utilized this technique to resolve the long-standing ambiguity regarding molecules that affect CggR interaction with DNA and serve as effectors. Previous biophysical and structural investigations have shown that various metabolites of the glycolytic pathway exhibit affinity for the CggR effector-binding site (13). This effector activity has been validated for fructose-1,6-bisfosfate (FBP) (16,22) and suggested for dihydroxyacetone phosphate (DHAP) (13). The K_D_ values for binding of FBP and DHAP to the isolated CggR effector-binding domain measured by isothermal titration calorimetry were 2.87 ± 0.06 μM and 3.34 ± 0.15 μM, respectively (13). Our cryo-EM study revealed complex disruption by FBP and also by DHAP resulting in the disassembly of the tetrameric structure into dimers, which either remain bound to the higher-affinity O_R_ or let go of the DNA completely. Previously published SAXS studies showed that upon the addition of FBP alone, the hydrodynamic properties of the CggR–DNA complex change, yet the protein remains loosely bound to the DNA as a tetramer (16). Considering the physical stress during the cryo-EM sample preparation, our studies cannot oppose these results. They, however, constitute evidence that one of the two dimers remains attached to the higher-affinity O_R_ even when subjected to such treatment.

The positive effect of FBP and DHAP is further supported by the results of our *in vitro* transcription assay, showing that transcription from the P*_CggR_* promoter gets switched on upon their addition. The transcription reached maxima at the concentration of DHAP of about 100 μM and FBP of 1 mM. It was reported that under non–glycolytic conditions, the intracellular concentration of DHAP and FBP in *B. subtilis* is about 0.5 and 0.2 mM, respectively (79), and thus the transcription of *gapA* operon should be inhibited. Indeed, our observations in *in vitro* transcription assay correspond to this range of values quite well.

For DeoR, the exact mechanism of release from DNA upon binding of the effector molecule deoxyribose-5-phosphate (dR5P) remains to be elucidated. It was previously shown that dR5P does not have any effect on the oligomeric state (14) and we confirmed presence of a tetramer bound to the operator even upon the binding of dR5P (Supplementary Figure S9C, D).

**Figure 6.**
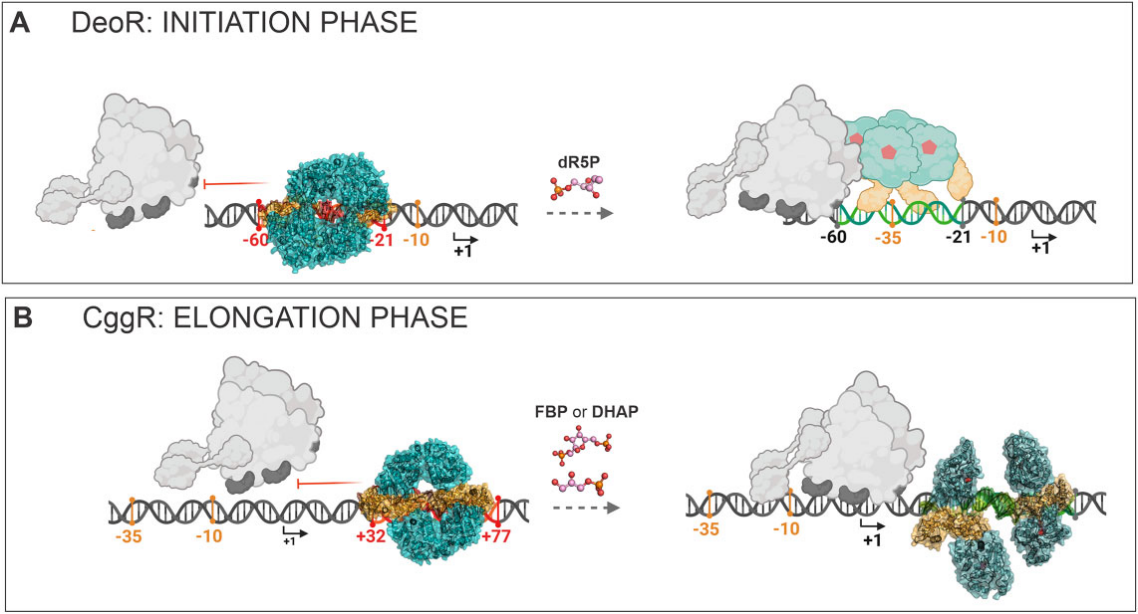
A schematic representation of the mechanism of action of SorC-family transcriptional repressors. (**A**) The here-presented DeoR-DNA model, which remains in the tetrameric form upon dR5P binding. The binding is probably not cooperative but the exact behavior and equilibria in the solution remain to be elucidated. (**B**) An expansion of the previously reported mechanism of CggR (16). We present here the exact architecture of the CggR–DNA complex and show DHAP to be the second effector molecule. The DBD is shown in shades of orange, the EBD in shades of teal and effector molecules in pink. The different color shades of the bound and unbound protein states indicate the conformational change. The DNA operator region is displayed in red and green in its repressed and de-repressed state, respectively. The numbers indicate the positions of the operator (in red) with respect to the transcription start point (in black; the arrows point in the direction of transcription) and the promoter elements −35 and −10 (in orange). The RNAP holoenzyme is shown in gray. The de-repressed state of DeoR is shown schematically without using the structure model because its behavior upon effector binding is not thoroughly known. The scheme was created using the BioRender.com tool.

In summary, we present the first structures of the SorC-family repressors in complexes with their cognate DNA operators, featuring two representative members of the SorC/DeoR and SorC/CggR subfamilies (Figure 6). Both model repressors share a common global tetrameric architecture when bound to DNA and similar structural response to an effector interacting with their EBDs. However, the CggR tetramer is disrupted into two dimers and DeoR, on the other hand, seems to maintain its tetrameric assembly. This persistence is probably balanced by the low affinity to the DNA, which allows the RNA-polymerase to read through the promoter.

This study provides valuable insight into the mechanism of the function of the two members belonging to the SorC family transcriptional regulators and serves as a foundation for further research on the biological function of this large family of bacterial regulators.

## Supplementary Material

gkae434_Supplemental_Files

## Data Availability

Atomic coordinates and structure factors or electron microscopy maps for the reported structures have been deposited with the Protein Data Bank and Electron Microscopy Data Bank under accession numbers 8R7Y (DeoR), and 8R3G and EMD-18864 (CggR).
