# Correction to ‘Structural characterization of two prototypical repressors of SorC family reveals tetrameric assemblies on DNA and mechanism of function’

**DOI:** 10.1093/nar/gkaf195

**Published:** 2025-03-07

This is a correction to: Markéta Šoltysová *et al.*, Structural characterization of two prototypical repressors of SorC family reveals tetrameric assemblies on DNA and mechanism of function, *Nucleic Acids Research*, Volume 52, Issue 12, 8 July 2024, Pages 7305–7320, https://doi.org/10.1093/nar/gkae434

In the version originally published, the Protein Data Bank entry for DeoR was given as 7Z6Y. This has been corrected to read 8R7Y.

