# Supplementary material for: Structural characterization of two prototypical repressors of SorC family reveals tetrameric assemblies on DNA and mechanism of function
Source: Nucleic Acids Res. 2024 Jun 6;52(12):7305–20. doi: 10.1093/nar/gkae434 (PMC11229326; doi:10.1093/nar/gkae434)
Supplement: gkae434_Supplemental_Files [file gkae434_supplemental_files.zip › R1_supplementary_final.pdf]

**SUPPLEMENTARY DATA**

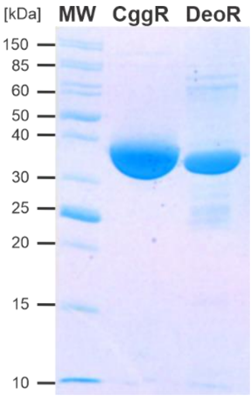


**Figure S1** Coomassie-blue stained SDS-PAGE gel of purified DeoR and CggR proteins. Unstained Proteins Standard, Broad Range,10-200 kDa (Biolabs, UK) was used as a molecular weight marker.

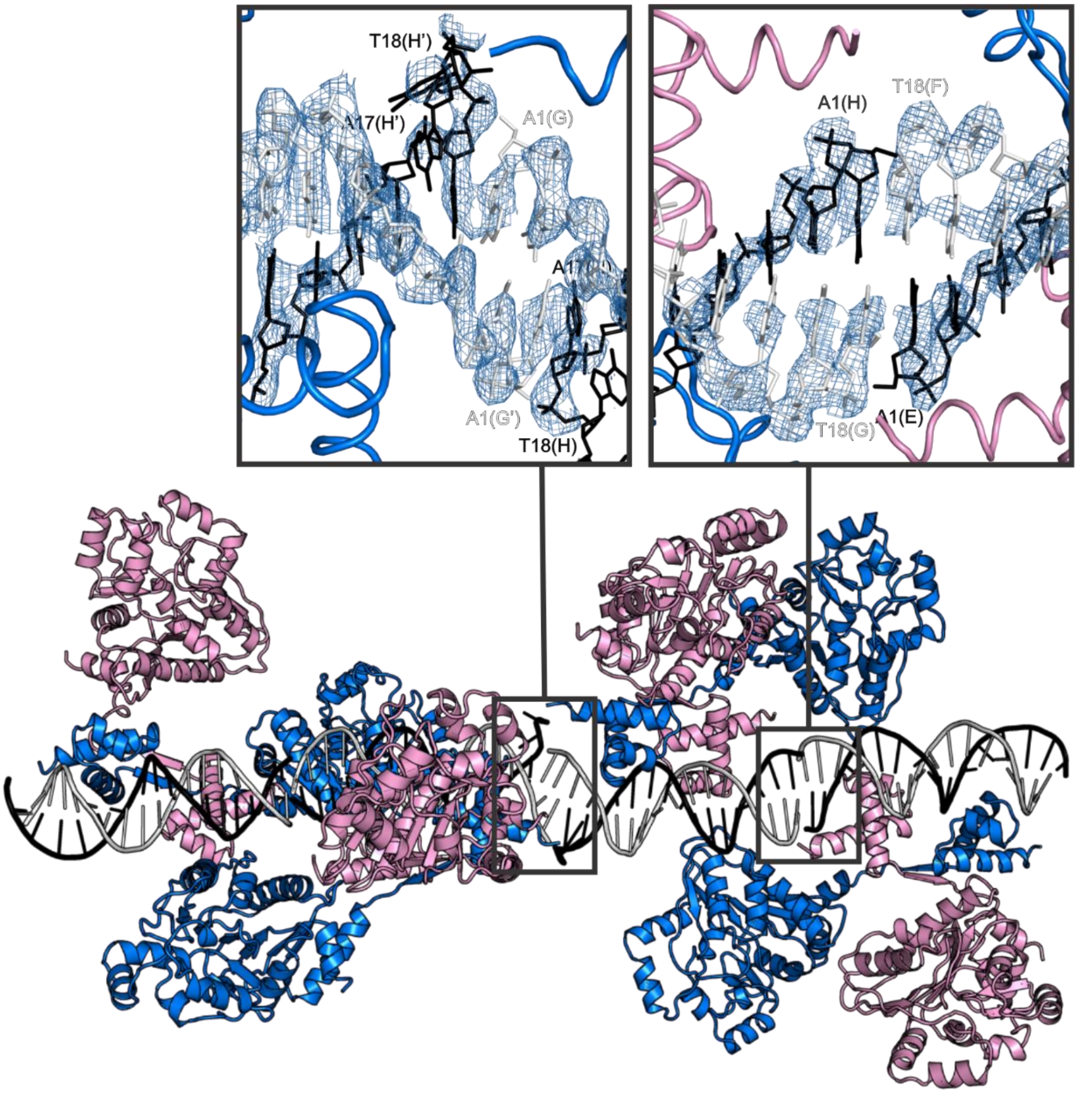


**Figure S2.** Head-to-tail packing of DNA duplexes in the DeoR–$O_{L18}$ crystal, resulting in a pseudo-continuous double helix and distorted DNA strands between the asymmetric units. A cartoon representation of two neighboring asymmetric units with a zoom-in view of the head-to-tail packing of the DNA double-strands within the asymmetric unit (right) and between two asymmetric units (left).

As mentioned in the main text, the major discrepancy between the DeoR–$O_{L18}$ and *bs*DeoR$_{DBD}$–$O_{15}$ models resides in the interaction pattern between R39 and the right side of the palindrome (chains A and D), which is likely a result of one base-pair deletion in $O_{15}$. In DeoR–$OL_{18}$, R39 in both dimers contacts the bases of T14 (strands F and G) and G4 (strands E and H). By contrast, R39 (chain B) in *bs*DeoR$_{DBD}$–$O_{15}$ interacts only with the phosphate backbone from the adjacent asymmetric unit. The side-chains of R39 in DeoR–$O_{L18}$ are not fully supported by the electron-density map, but such interactions are highly plausible, judging also by the resulting interaction symmetry with the left palindrome.

Another noteworthy difference lies in the interaction pattern between R34 and the right side of the palindrome. In DeoR–$O_{L18}$, R34 (chains A and D) forms a hydrogen bond with the base of T12 (strands F and G), which is a nucleotide of the TA base-pair missing in the *bs*$DeoR_{DBD}$–$O_{15}$ model, in which R34 interacts with the adjacent adenine instead. The side chain of R34 in DeoR–$O_{L18}$ is supported by the electron density map only in chain D.

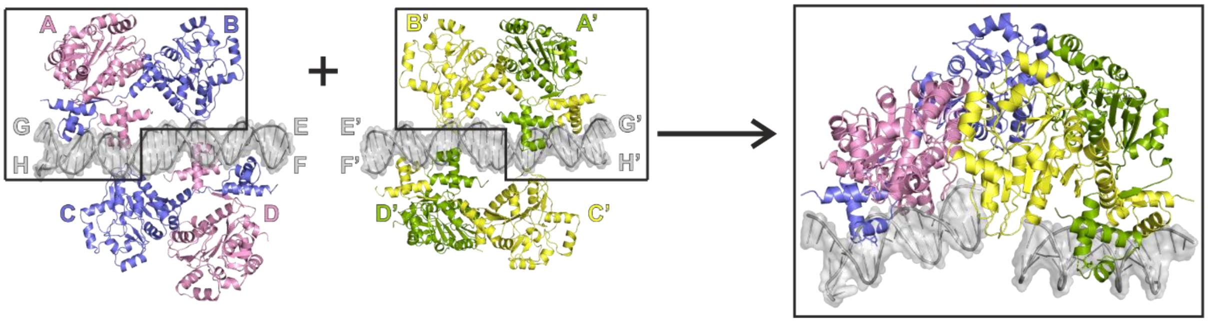


**Figure S3.** Reconstruction of the DeoR–$O_{L18}$ biological unit from the crystal structure. The asymmetric unit comprises two independent dimers (chains AB and CD), each bound to one DNA duplex $O_{L18}$ (chains GH and EF, respectively). DeoR dimers of the basic asymmetric unit and the symmetry-related DeoR dimers are distinguished by pink and blue (AB and CD) and green and yellow (A′B′ and C′D′), respectively. The symmetry-related dimeric DeoR–$O_{L18}$ complexes forming the biological unit are highlighted in black frames (left), as is the resulting biological unit (right).

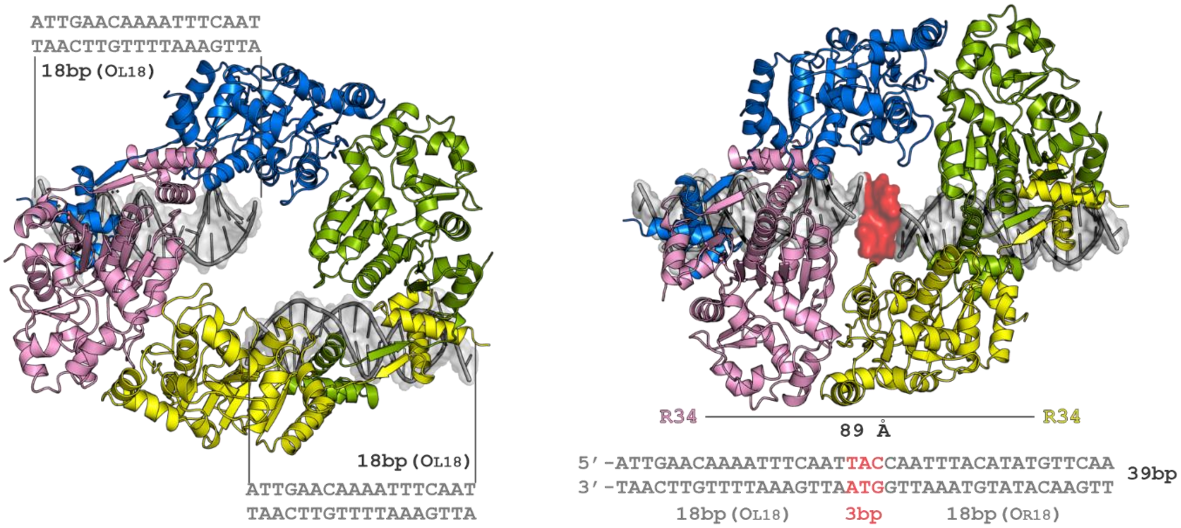


**Figure S4.** Reconstruction of the DeoR tetramer model bound to a continuous DNA double-strand. The added 3 bp region is highlighted in red in the right cartoon representation.

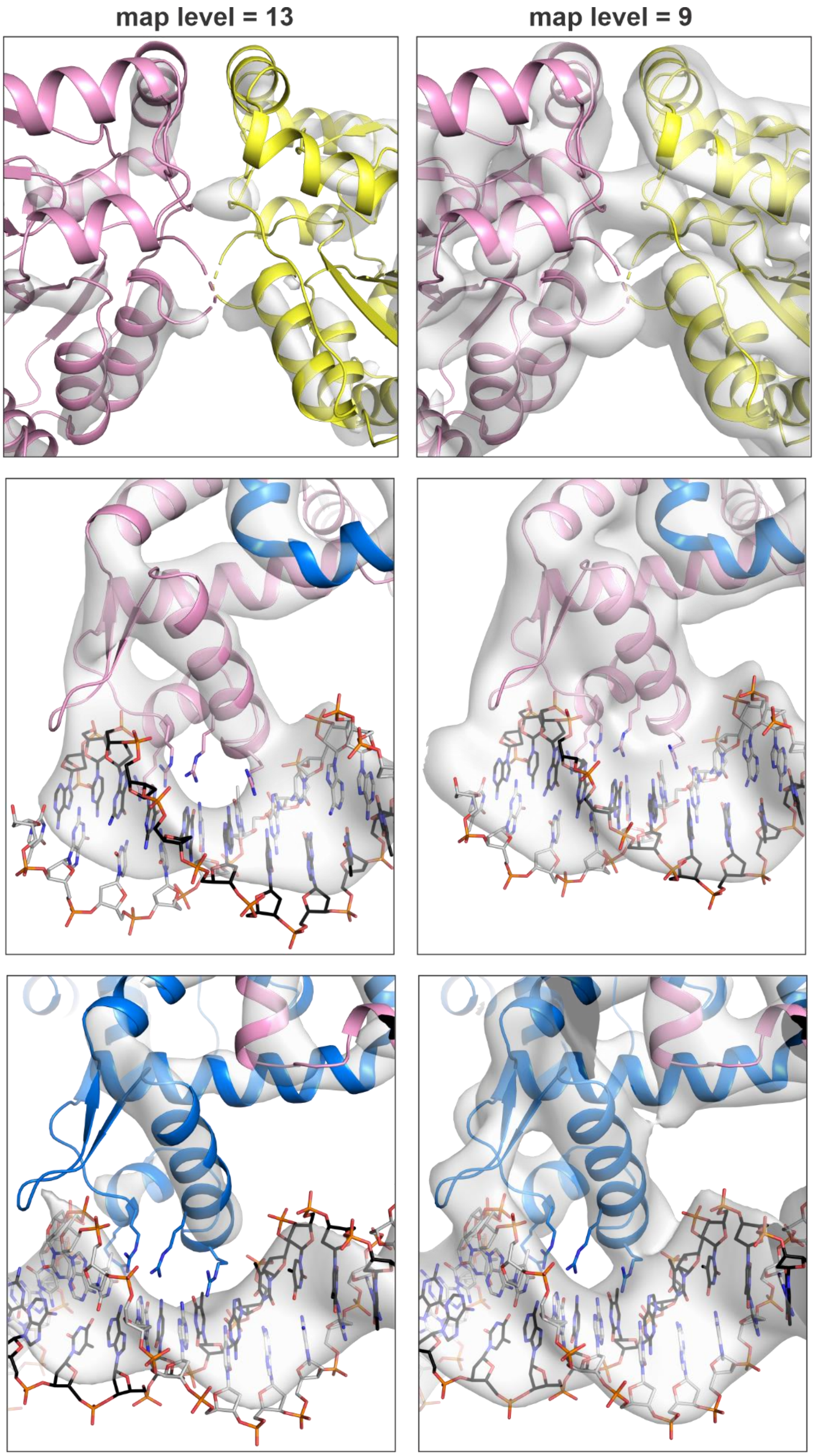
map level = 13
map level = 9

**Figure S5.** Detail on the cryo-EM map of EBD-EBD and DBD-DNA interfaces at electron density (contour) levels of 13 and 9 indicated above the corresponding panels.

−35 −10 TSS *bs*CggR binding site

CAGTTGAATAAACAATTCCACCCTGTTAAAATAATTAAAGAAAGCAGAAATGATTTTTTTTGGCTATGACGGGACGTTTTTTGTCATAGCGGGACATATAATGTCCAGCAAAAAAGGAAGGAACGTTTGAGTC

**Figure S6.** The $P_{CggR}$ promoter. Conserved −35 and −10 hexamers are labeled and highlighted in green, the transcription start sites (TSS) are shown in red, and the CggR binding site is shown in violet.

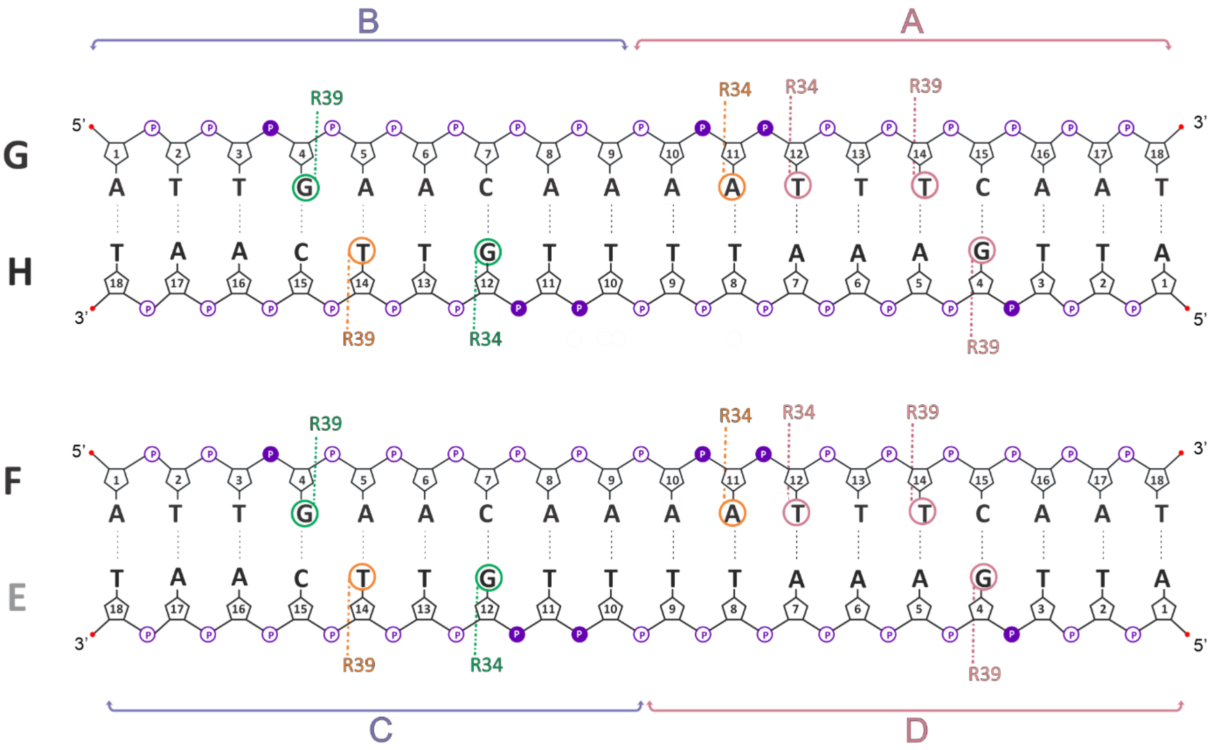


**Figure S7.** Schematic representation of the DeoR–$O_{L18}$ probable base-specific hydrogen bonding. The schemes are based on PISA analysis (47) and manual inspection was generated by the NUCPLOT software (75) and adjusted. Both DNA duplexes present in the asymmetric unit (strands GH and FE) are shown. Amino-acid residues of chains A and D are in pink, and chains B and C are in blue. To indicate major differences between DeoR–$O_{L18}$ and *bs*DeoR$_{DBD}$–$O_{15}$ (7BHY), all base-specific interactions identical in both structures are shown in green, the interactions that occur only in the *bs*DeoR$_{DBD}$–$O_{15}$ model are shown in orange, and the possible interactions identified only in the DeoR-$O_{L18}$ structure are shown in the color of the respective chain (pink). The phosphate groups contacted by the protein in the DeoR–$O_{L18}$ structure are highlighted by filled purple circles.

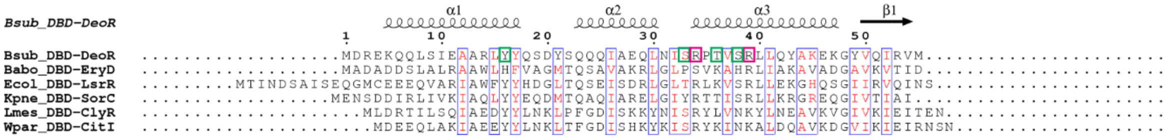


**Figure S8.** A sequence alignment of selected SorC / DeoR DBDs. The conserved arginines which are supposed to be involved in base readout are highlighted in pink rectangles. The residues known to interact with phosphate backbone are in green rectangles.

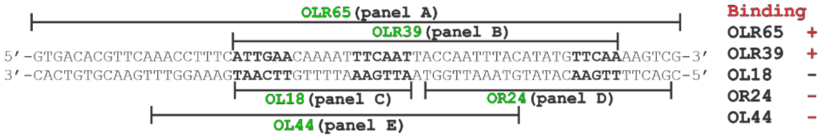


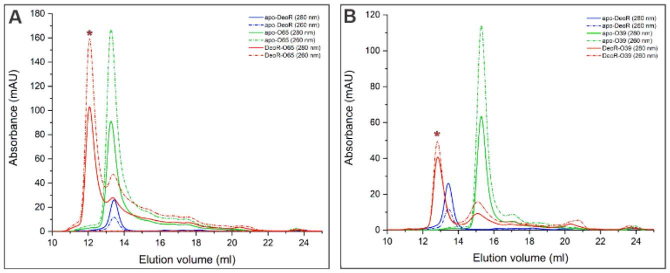

| Sample | apo-DeoR | DeoR-OLR65 |
|---|---|---|
| Theoretical $MW_{theor}$ | 35.7 kDa/142.9 kDa (monomer/tetramer) | 182.9 kDa (tetramer + DNA) |
| Elution volume | 13.44 ml | 12.07 ml |
| Apparent $MW_{app}$ [a] | 142.5 kDa | 272.3 kDa |
| Assembly[b] | tetramer (4.0) | tetramer + DNA (1.5) |

| Sample | apo-DeoR | DeoR-OLR39 |
|---|---|---|
| Theoretical $MW_{theor}$ | 35.7 kDa/142.9 kDa (monomer/tetramer) | 166.9 kDa (tetramer + DNA) |
| Elution volume | 13.44 ml | 12.84 ml |
| Apparent $MW_{app}$ [a] | 142.5 kDa | 189.2 kDa |
| Assembly[b] | tetramer (4.0) | tetramer+DNA (1.1) |

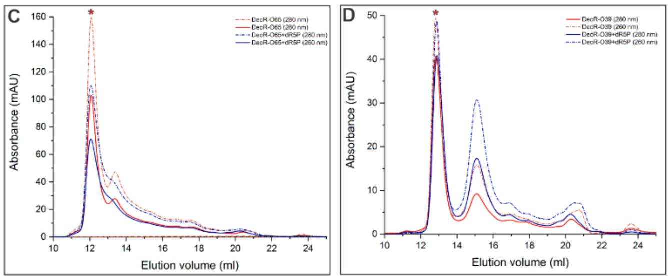

| Sample | DeoR-OLR65 | DeoR-OLR65 + dR5P |
|---|---|---|
| Theoretical $MW_{theor}$ | 182.9 kDa (tetramer + DNA) | 182.9 kDa (tetramer + DNA) |
| Elution volume | 12.07 ml | 12.06 ml |
| Apparent $MW_{app}$ [a] | 272.3 kDa | 273.6 kDa |
| Assembly[b] | tetramer + DNA (1.5) | tetramer + DNA (1.5) |

| Sample | DeoR-OLR39 | DeoR-OLR39 + dR5P |
|---|---|---|
| Theoretical $MW_{theor}$ | 166.9 kDa (tetramer + DNA) | 166.9 kDa (tetramer + DNA) |
| Elution volume | 12.84 ml | 12.90 ml |
| Apparent $MW_{app}$ [a] | 189.2 kDa | 184.0 kDa |
| Assembly[b] | tetramer+DNA (1.1) | tetramer+DNA (1.1) |

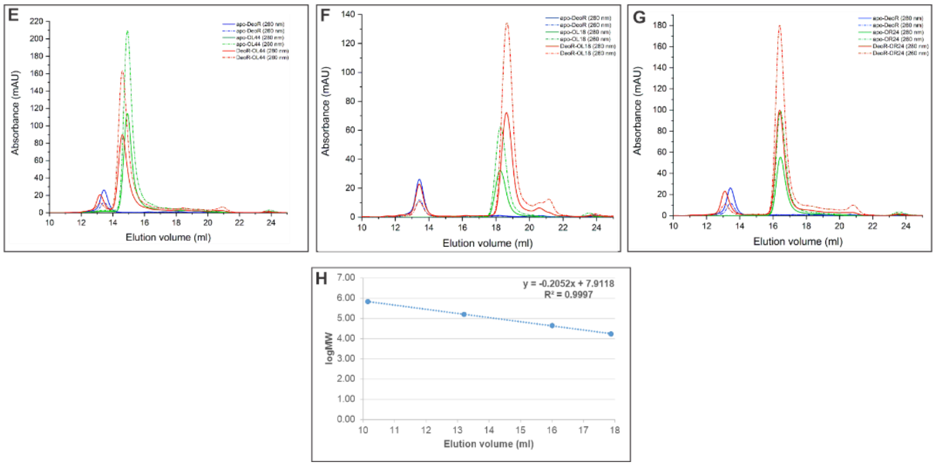

[a]$MW_{app} = 10^{(-0.2052 \times Ve + 7.9118)}$, [b]Assembly = $MW_{app}/MW_{theor}$(monomer or tetramer-DNA complex)

**Figure S9.** Size-exclusion chromatography analysis of DeoR and DeoR–DNA complexes. (A, B, E –G). Overlays of chromatograms at 280 and 260 nm of apo-DeoR, single oligonucleotides and DeoR–DNA mixtures. The red asterisks indicate signals of the DeoR–DNA complexes. (C, D) Overlays of chromatograms at 280 and 260 nm of DeoR-DNA complexes and their mixtures with dR5P effector. (H) The calibration of the Superdex 200 10/300 GL column. The upper part of the figure presents the tested oligonucleotides in the context of the operator region DNA sequence. The “+” and “−” symbols indicate whether the respective oligonucleotide forms a complex with DeoR or not, respectively. The palindromic sequences and the direct repeat are in bold.

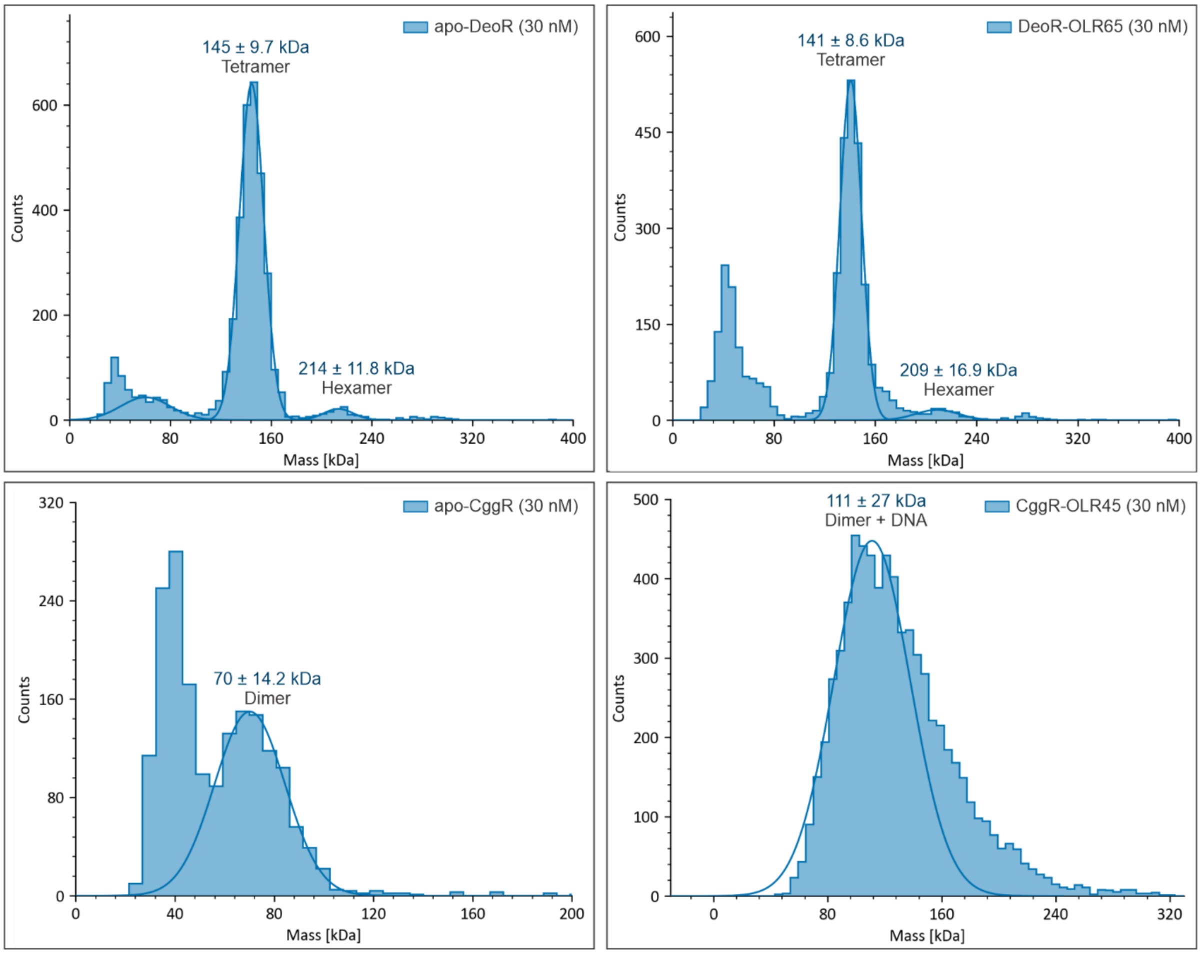


**Figure S10.** Molecular mass distribution histograms of DeoR and CggR of their apo-forms and complexes with DNA at the final concentration of 30 nM. Free DeoR forms tetramers while apo-CggR was measured was represented by a single population of dimers. The analysis of DeoR with the full operator $O_{LR}$ (65 bp) revealed tetramer, CggR in complex with the full operator $O_{LR}$ (45 bp) complex revealed a mass population corresponding to a dimer bound to the DNA. At the given concentration (30 nM CggR), the result goes in perfect agreement with the previous studies showing a 100-fold higher affinity of CggR for $O_R$ ($K_D$ < 0.5 nM) than for $O_L$ ($K_D$ = 50 nM) (71).

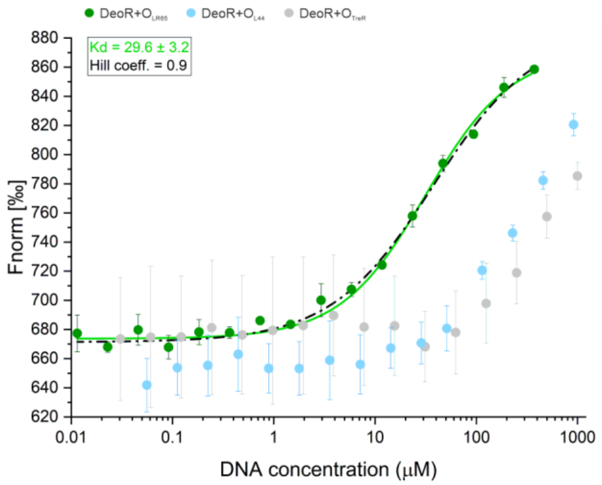


**Figure S11.** MST analysis of DeoR-DNA interaction. $O_{LR65}$ (green), $O_{L44}$ (blue) and negative control (grey; an unrelated 46 bp-long DNA operator of transcriptional repressor TreR) are shown. Data from the DeoR-$O_{LR65}$ measurement were used to derive the binding curve (green line), binding isotherm (black dot-dash line), $K_D$ and Hill coefficient. The data of the DeoR-$O_{LR65}$ analysis were selected from two independent measurements, which were carried out in three triplicates. The binding curve and isotherm represent the mean curves derived from the measurements.

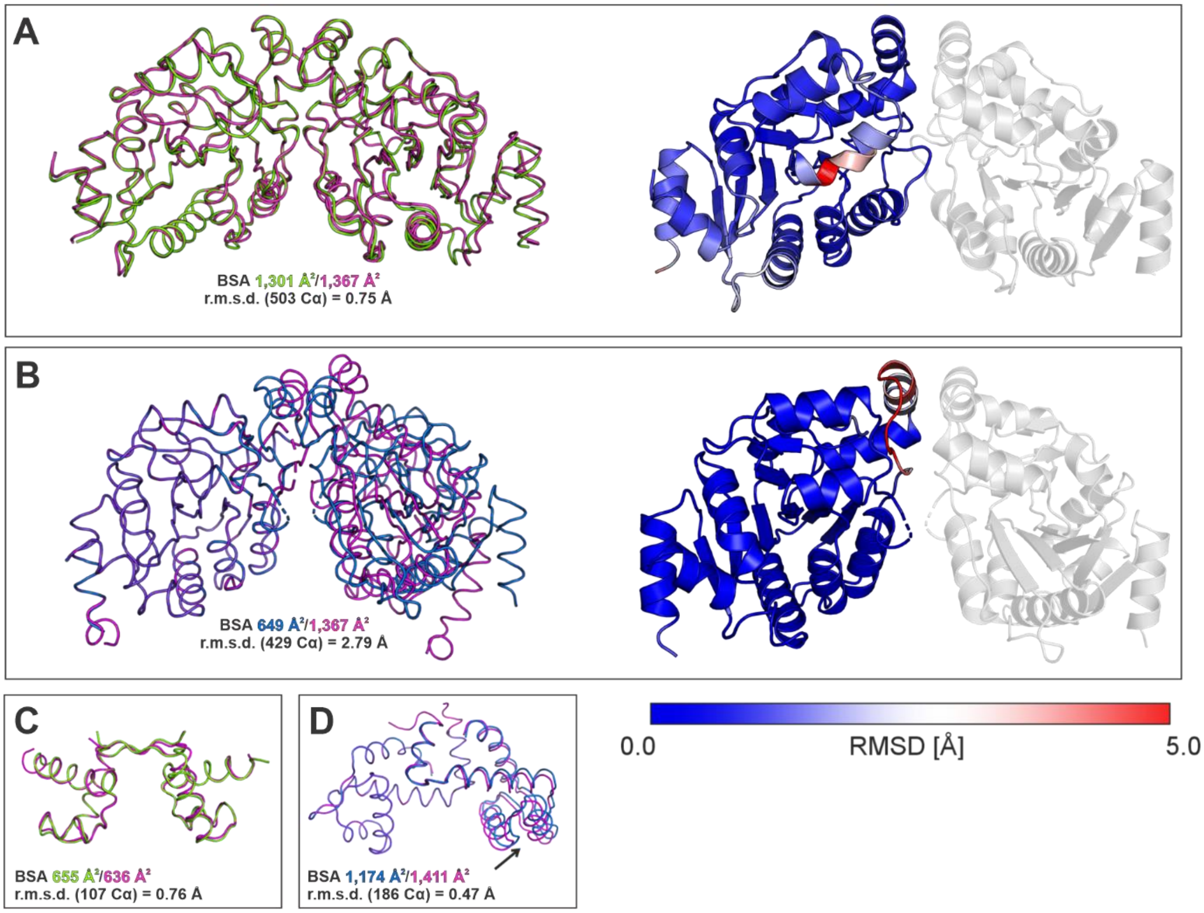


**Figure S12.** Comparison of EBD and DBD dimers in our full-length models compared to previously published EBD and DBD structures. The EBD and DBD dimers are superposed over monomers (shown in a cartoon loop representation on the left side). On the right side, the superposed EBD monomers are colored based on differences in r.m.s.d. values. The r.m.s.d. scale is provided at the bottom. (A) EBD of DeoR (green) and 4OQQ (magenta) (14). (B) EBD of CggR (blue) and 2OKG (magenta) (13). (C) Left: DBD of DeoR (green) and 7BHY (magenta) (23). Right: DBD of CggR–$O_{L18}$ (blue) and 7OYK (magenta) (23). The black arrow indicates the more open conformation of the DBD dimer in the full-length CggR model.

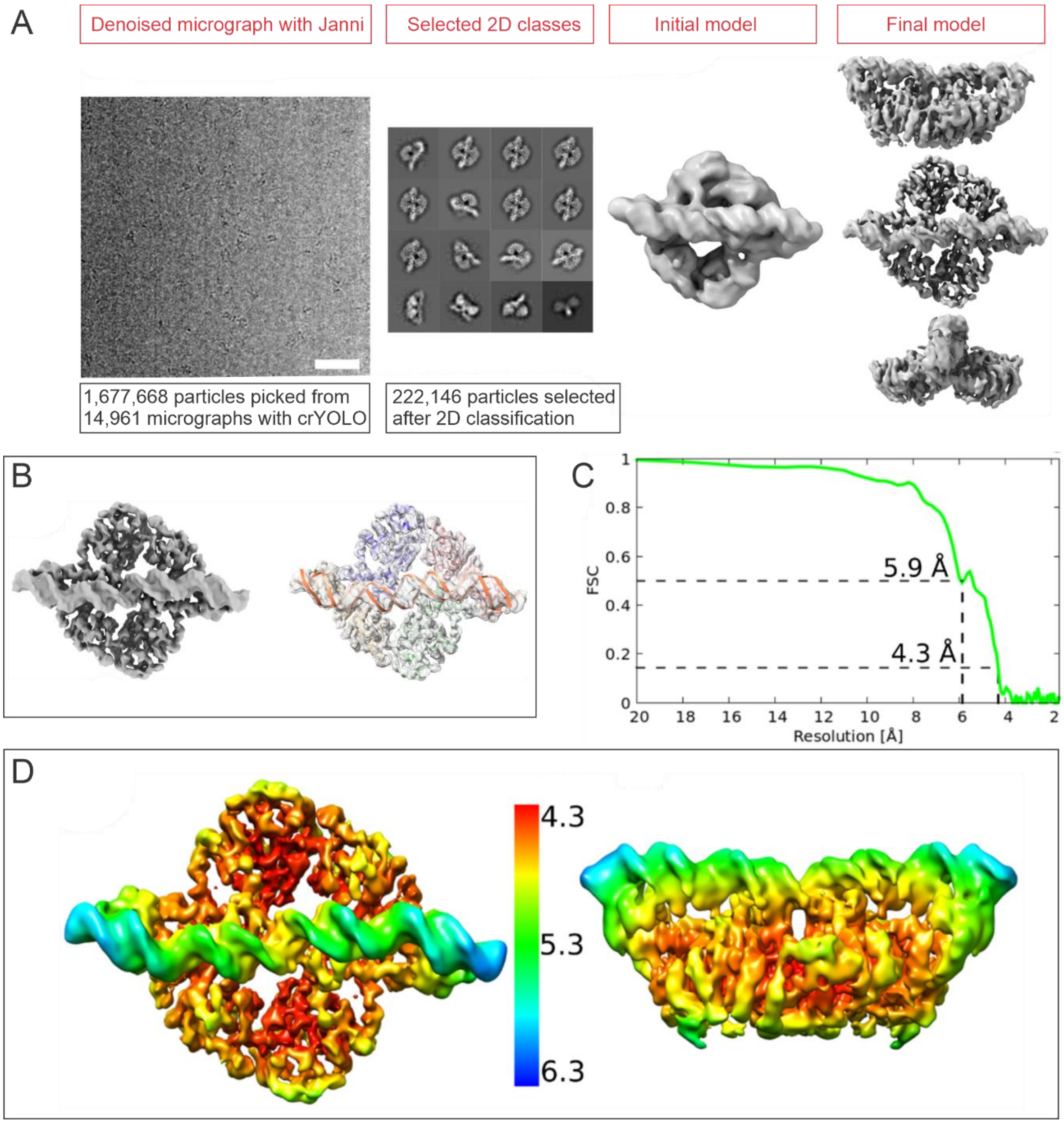


**Figure S13.** Data processing and the final cryo-EM map of the CggR-O<sub>LR</sub> complex. (A) Data processing procedure for the CggR–O<sub>LR</sub> using RELION3.1 (57) and CRYOSPARC (58). The final cryo-EM map was determined from 220,146 particles and reached a global resolution of 4.3 Å according to the FSC 0.143 criterion. The scale bar in the micrograph corresponds to 50 nm. (B) Cryo-EM map as viewed along the two-fold symmetry axis (left) and the fit of the CggR tetramer and DNA into the cryo-EM map (right). (C) Fourier shell correlation (FSC) plot for the refinement of the CggR–O<sub>LR</sub> cryo-EM data with the overall resolution highlighted for FSC 0.5 and FSC 0.143, respectively. (D) Local resolution assessment of the final cryo-EM map.

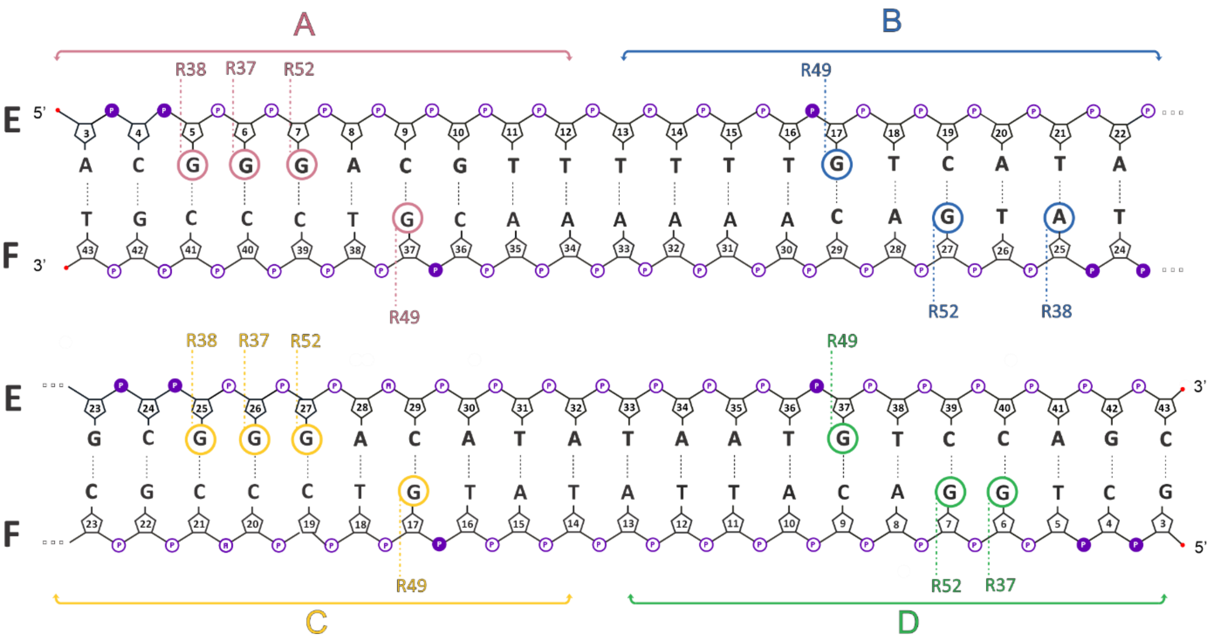


**Figure S14.** Schematic representation of potential CggR–$O_{LR}$ base-specific hydrogen bonding. The scheme was generated by NUCPLOT software (75) based on manual evaluation of potential interactions. Amino-acid residues from chains A, B, C and D are in pink, blue, yellow and green, respectively. The phosphate groups contacted by the protein are highlighted as filled purple circles.

**Table S1.** Oligonucleotides used for the SEC-based DeoR–DNA binding studies and the 34 bp oligonucleotide used in the study by Zeng and Saxild (labeled with an asterisk) (25).

| **DNA** | **Sequence** | **bp** |
|---|---|---|
| $O_{LR65}$ | 5′ – GTGACACGTTCAAACCTTTCATTGAACAAAATTTCAATTACCAATTTACATATGTTCAAAAGTCG<br>3′ – CACTGTGCAAGTTTGGAAAGTAACTTGTTTTAAAGTTAATGGTTAAATGTATACAAGTTTTCAGC | 65 |
| $O_{LR39}$ | 5′ – ATTGAACAAAATTTCAATTACCAATTTACATATGTTCAA<br>3′ – TAACTTGTTTTAAAGTTAATGGTTAAATGTATACAAGTT | 39 |
| $O_{L44}$ | 5′ – CACGTTCAAACCTTTCATTGAACAAAATTTCAATTACCAATTTACA<br>3′ – GTGCAAGTTTGGAAAGTAACTTGTTTTAAAGTTAATGGTTAAATGT | 44 |
| $O_{L18}$ | 5′ – ATTGAACAAAATTTCAAT<br>3′ – TAACTTGTTTTAAAGTTA | 18 |
| $O_{R24}$ | 5′ – TACCAATTTACATATGTTCAAAAG<br>3′ – ATGGTTAAATGTATACAAGTTTTC | 24 |

**Table S2.** DNA variants used for the crystallization of the DeoR–DNA complexes. Base symbols highlighted in color stand for the following: green – additional bases that agree with the native sequence; red – alternative bases with assumable higher potential to stabilize the duplex. The asterisk symbol (*) denotes the DNA variant designed based on the right half of the operator. The square symbols (□) denote excluded base pairs.

| DNA | Sequence | bp |
|---|---|---|
| $O_{LR39}$ | 5′ – ATTGAACAAAATTTCAATTACCAATTTACATATGTTCAA<br>3′ – TAACTTGTTTTAAAGTTAATGGTTAAATGTATACAAGTT | 39 |
| $O_{2x17}$ | 5′ – ATTGAACAAAATTTCAA□ATTGAACAAAATTTCAA<br>3′ – TAACTTGTTTTAAAGTT□TAACTTGTTTTAAAGTT | 34 |
| $O_{32}$ | 5′ – ATTGAACAAAA□TTCAA□ATTGAACAAAA□TTCAA<br>3′ – TAACTTGTTTT□AAGTT□TAACTTGTTTT□AAGTT | 32 |
| $O_{30}$ | 5′ – ATTGAACAAAA□TTCAA□□TTGAACAAAA□TTCAA<br>3′ – TAACTTGTTTT□AAGTT□□AACTTGTTTT□AAGTT | 30 |
| $O_{20a}$ | 5′ – **C**ATTGAACAAAATTTCAATT<br>3′ – **G**TAACTTGTTTTAAAGTTAA | 20 |
| $O_{20b}$ | 5′ – **C**ATTGAACAAAATTTCAAT**C**<br>3′ – **G**TAACTTGTTTTAAAGTTA**G** | 20 |
| $O_{21}$ | 5′ – **C**ATTGAACAAAATTTCAATT**C**<br>3′ – **G**TAACTTGTTTTAAAGTTAA**G** | 21 |
| $O_{22}$ | 5′ – **TC**ATTGAACAAAATTTCAATT**C**<br>3′ – **AG**TAACTTGTTTTAAAGTTAA**G** | 22 |
| $O_{24a}$ | 5′ – **TTC**ATTGAACAAAATTTCAATTAC<br>3′ – **AAG**TAACTTGTTTTAAAGTTAATG | 24 |
| $O_{24b}$ | 5′ – **CTTC**ATTGAACAAAATTTCAATT**G**<br>3′ – **GAAG**TAACTTGTTTTAAAGTTAA**C** | 24 |
| $O_{L18}$ | 5′ – ATTGAACAAAATTTCAAT<br>3′ – TAACTTGTTTTAAAGTTA | 18 |
| $O_{18}$ overhang | 5′ – ATTGAACAAAATTTCAATT**G**<br>3′ – A**C**TAACTTGTTTTAAAGTTA | 20 |
| $O_{17a}$ | 5′ – ATTGAACAAAATTTCAA<br>3′ – TAACTTGTTTTAAAGTT | 17 |
| $O_{17b}$ | 5′ – TTGAACAAAATTTCAAT<br>3′ – AACTTGTTTTAAAGTTA | 17 |
| $O_{17c}$* | 5′ – AATTTACATATGTTCAA<br>3′ – TTAAATGTATACAAGTT | 17 |
| $O_{16+1}$ | 5′ – TTGAACAAAATTTCAA<br>3′ – TAACTTGTTTTAAAGTT | 16/17 |
| $O_{16a}$ | 5′ – ATTGAACAAAATTTCA<br>3′ – TAACTTGTTTTAAAGT | 16 |
| $O_{16b}$ | 5′ – ATTGAACAAAA□TTCAA<br>3′ – TAACTTGTTTT□AAGTT | 16 |
| $O_{15+2}$ | 5′ – TGAACAAAATTTCAA | 15/17 |

| | | |
|---|---|---|
| | 3′ - TAACTTGTTTTAAAGTT | |
| $O_{15a}$ | 5′ - TTGAACAAAATTTCAA<br>3′ - AACTTGTTTTAAAGTT | 15 |
| $O_{15b}$ | 5′ - TTGAACAAAA□TTCAA<br>3′ - AACTTGTTTT□AAGTT | 15 |
| $O_{15}$ overhang | 5′ - TGAACAAAATTTCAA<br>3′ - TAACTTGTTTTAAAG | 15 |
